# Changes in Protonation State of Atmospherically Relevant α‑Hydroxyacids at the Air–Water Interface Measured by Surface Tension and IR-RAS

**DOI:** 10.1021/acs.jpca.5c02825

**Published:** 2025-07-29

**Authors:** Burgess E. Rugeley, Katherine R. Holt, Erica B. Peterson, Selma Moulai-Khatir, Claire F. N. Koltun, Rebecca J. Rapf

**Affiliations:** 7077 Trinity University, Department of Chemistry, San Antonio, Texas 78212, United States

## Abstract

Characterization of the acid–base behavior and surface protonation state of atmospherically relevant organic acids is of key importance in our understanding of interfacial reactivity, as well as our ability to accurately model aerosol impact on climate. Here we investigate the protonation state of two medium-chain α-hydroxyacids, 2-hydroxyhexanoic acid (HHA) and 2-hydroxyoctanoic acid (HOA), at the air–water interface and in the bulk. The ratio of surface-deprotonated to surface-protonated species at varying pH was examined using surface tension titrations, finding an effective surface-p*K*_a_ of 4.5 ± 0.2 for HHA and 5.41 ± 0.05 for HOA, both of which are significantly higher than their bulk p*K*_a_ values of 3.9 ± 0.1 and 4.0 ± 0.1, respectively, which were determined *via* potentiometric titration. However, the effective surface-p*K*_a_ obtained from surface tension measurements also contains contributions from adsorption and desorption processes, which means that it does not directly probe differences in the dissociation equilibrium at the interface. We show that infrared reflection–absorption spectroscopy (IR-RAS) can be used to directly probe the surface dissociation of α-hydroxyacids *in situ* for the first time, demonstrating the utility of IR-RAS as a technique for these types of studies. By correcting for the relative surface activity of the anion and acid species, the surface-p*K*_a_ obtained using IR-RAS is a better measure of the actual shift in dissociation equilibrium at the interface. Through comparison to the bulk spectra obtained using attenuated total reflectance (ATR) spectroscopy, we confirmed that the protonated form of the α-hydroxyacids is favored at the water surface. However, we find that the difference between the surface-p*K*_a_ and bulk pK_a_ obtained spectroscopically is 0.2 ± 0.1 for HHA and 0.4 ± 0.2 for HOA. This suggests that the relative shift in the dissociation constant at the interface is modest, and that adsorption processes play an important role in the speciation at the interface and must be explicitly considered in these studies. Overall, we confirm the importance of fundamental lab studies to examine the speciation at air–water interfaces as a function of solution condition, as bulk pH alone is not sufficient to predict the distribution of species present at the interface.

## Graphical abstract

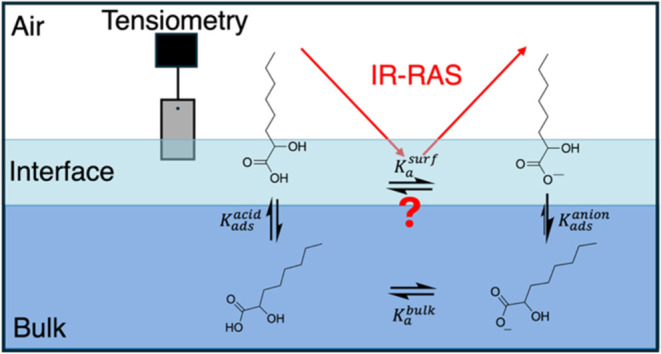

## 1 Introduction

Observations of changes in reactivity at air–water interfaces and in confined microenvironments have been the subject of considerable recent interest in the literature.^1–6^ Such air–water interfaces are ubiquitous in natural environments, including at the surface of oceans and on atmospheric aerosol. These surfaces concentrate organic molecules because even highly soluble organic species partition to air–water interfaces. The resultant interfacial organic films affect the physical, chemical, and optical properties of aerosol,^7–13^ influencing their contributions to radiative forcing and, ultimately, climate. Therefore, understanding the interfacial behavior of environmentally relevant molecules, including organic acids, is crucial for predicting the reactivity of these species under atmospheric conditions.

Alkyl organic acids are important environmentally relevant surface-active species that are found in the sea surface microlayer and on atmospheric aerosol.^11,14–20^ These include fatty acids, as well as other substituted carboxylic acids, such as α-keto acids, α-hydroxyacids, and anthropogenic perfluorinated acids. These organic acids span a wide range of size and surface activity from highly soluble species (C_2_–C_3_) to insoluble species with long alkyl chains (C_16_–C_18_ or larger),^19–23^ but organic acids with intermediate alkyl tail length (C_6_–C_10_) are of particular interest because they act as soluble surfactants, with both a high surface propensity as well as a significant bulk solubility. The surface activity of these “medium-chain” organic acids is also highly pH dependent, as the protonated, uncharged acid form is more surface active than the corresponding deprotonated, charged anion.^24–27^ Characterizing the protonation state of these medium-chain organic acids at the interface is critical to elucidate both how pH affects the properties of the aerosol surface, as well as which species are available at the water surface to participate in interfacial reactivity.

It has been widely shown that the ionization state of organic species is different at the water surface than observed in the bulk. In general, studies have shown that neutral species are favored at the interface,^25–51^ although this trend has not been universally observed.^52–55^ For acids, this means that a lower degree of dissociation is expected at the surface. This shift to lower deprotonation at interfaces has been reported for fatty acids,^25,26,40–47^ as well as substituted carboxylic acids, including α-keto acids,^28^ amino acids,^48^ and fluorinated acids.^38^ For fatty acids, the magnitude of this shift to favor the protonated acid at the interface has been observed to increase with increasing alkyl tail length.^26,40–46^ However, shifts in protonation state have been found to occur even for highly soluble species, including pyruvic acid.^28^

A wide-range of experimental techniques have been used to characterize ionization state at the air–water interface, including acid–base titrations,^41–43^ X-ray photoelectron spectroscopy,^46,47,56^ electrospray ionization mass spectrometry,^52,55^ and surface-sensitive vibrational spectroscopy,^28,30,33,36,37,48,49,57–59^ among others. For soluble alkyl organic acids, surface tension titrations are a well-established technique to determine the protonation state of species at the air–water interface.^26,38,60^ The presence of organic species at the interface, whether protonated or deprotonated, lowers the surface tension relative to that of pure water. In surface tension titration studies,^26^ a dilute (∼mM) solution of the organic acid of interest is typically adjusted to a high starting pH (∼12) with NaOH, and then HCl is used as the titrant to lower the pH. The surface tension is measured at each point in the titration. At low pH, when the acid is protonated, more molecules are present at the interface, resulting in a lower surface tension. At high pH, the acid exists in its deprotonated anion form, which is more soluble and, therefore, less surface active, which results in a higher surface tension. The surface tension measurements can then be fit to the following surface activity model^26^

1 $$\Delta \gamma =\frac{\Delta \gamma_{\max}}{1+10^{\left({\mathrm{pH}}_{b}-{\mathrm{pKa}}_{s}\right)}}$$

where Δγ is the change in surface tension relative to the maximum surface tension value (γ_max_, obtained at high pH), given by Δγ = γ_max_ – γ. The maximum change in surface tension over the titration, Δγ_max_, is obtained at low pH. pH_b_ is the pH of the bulk solution and p*K*_as_ is a fitting parameter representing the “surface-pKa” of the solution. Based on this model, it has been shown that fatty acids favor the neutral acid form at the air–water interface, with nonanoic acid, for example, having a reported “surface-pKa” of 5.8 ± 0.1,^26^ an increase of ∼0.8 pH units from the bulk p*K*_a_ of nonanoic acid (4.96).^61^

While it is standard in the field to refer to the fit parameter of this surface activity model as the “surface-pKa”,^26,38,60^ this does not, in fact, represent the true dissociation equilibrium for the acid at the interface (*K*_a_^surf^), as can be seen in Figure 1. One of the key assumptions underlying this surface activity model is that the anion has a lower surface activity than the acid. The protonated forms of organic acids are more surface active, while the deprotonated, charged anion forms have a larger solubility in the bulk (*K*_ads_^acid^ > *K*_ads_^anion^).^24–27^ It is assumed that at high pH (when only the anion is present) the majority of molecules have been solubilized and are no longer present at the water surface, given the low concentrations of soluble surfactants that are used in these surface tension titrations. Indeed, it has been observed that long equilibration times are required for the stabilization of surface tension measurements at intermediate pH conditions (up to ∼20 min),^26^ suggesting that diffusion and adsorption processes play an important role in speciation at the interface. The importance of considering such processes has been previously addressed by others,^25,51^ including by Luo et al.^25^ who showed that differences in surface activity between the acid and anion can account for the shift in “surface-pKa” for nonanoic acid that has been determined from surface tension measurements. This means that surface tension measurements alone cannot measure the true surface dissociation equilibrium (*K*_a_^surf^) because surface adsorption and desorption are also occurring at the same time, and the fitting parameter used in the surface activity model includes contributions from both.^25^

**Figure 1:**
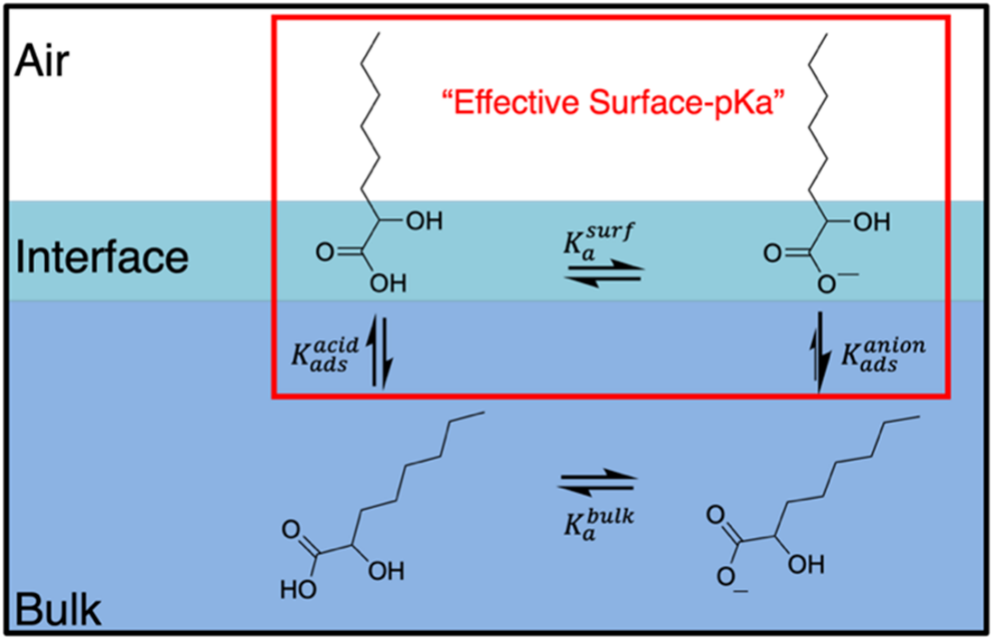
Cartoon schematic of the equilibria governing the dissociation of α-hydroxyoctanoic acid at different pH conditions, inspired by the representation given in Luo et al.,^25^ showing the true surface dissociation *(K*_a_^surf^), the bulk dissociation (*K*_a_^bulk^), and the adsorption equilibrium for both the acid (*K*_ads_^acid^) and anion (*K*_ads_^anion^), where the larger arrow denotes that the anion is significantly less surface active than the acid. The red box represents the coupled processes included in the “effective surface-pKa” value obtained by fitting surface tension data to a surface activity model.

Despite these limitations, surface tension measurements can give highly useful insight into the ratio of surface-deprotonated to surface-protonated species at varying pH, and the “surface-pKa” value obtained from fitting this data to the surface activity model gives a useful quantitative metric for comparison between molecules. We will use the term “effective surface-pKa” throughout the rest of the manuscript to refer to this fitting parameter, noting that it is a simplification of the entangled dissociation and adsorption processes occurring at the interface.

While surface tension measurements provide an understanding of the distribution of species present at the interface, they cannot explicitly probe whether surface dissociation is different from the bulk. Computational studies can be used to examine shifts in surface dissociation,^25,29,35,40^ but it has proven challenging to obtain reliable experimental measurements that are specific to the surface dissociation process for soluble acids.^25,48,51,62^ Recently, surface-sensitive spectroscopic techniques have been shown to be a promising tool for these types of studies, and vibrational sum frequency generation spectroscopy has been used to investigate the degree of dissociation of soluble organic acids at the interface.^28,48^ Here, we will show that infrared reflection–absorption spectroscopy (IR-RAS) can also be used as a complementary and more accessible spectroscopic method for such studies.

IR-RAS is a linear spectroscopic technique that has been previously shown to provide surface-specific spectra for soluble organic acids^63^ and has been used to examine a wide-range of alkyl organic acids, including medium-chain fatty acids,^25,26^ α-keto acids,^64,65^ and α-hydroxyacids.^63,66^ Although it has been previously used to examine the surface protonation of long-chain, insoluble organic acids,^37,57–59^ studies examining the surface protonation state of medium-chain fatty acids have used IR-RAS solely to confirm the relative surface activity of molecules by focusing solely on the C–H region of the vibrational spectrum.^25,26^ While qualitative differences in the carbonyl region for phenylalanine,^54^ α-keto acids,^64,65^ and α-hydroxyoctanoic acid^66^ have been reported, to the best of our knowledge, we show here for the first time that IR-RAS can be used to directly examine the protonation state of soluble organic acids *in situ*.

Here, we focus on medium-chain α-hydroxyacids, a class of environmentally relevant substituted fatty acids. As α-hydroxyacids differ from fatty acids by only the presence of a hydroxyl group, they allow for a detailed comparison to the better studied carboxylic acids and give insight into the importance of molecular specificity in understanding the behavior and reactivity of organic species at interfaces. We use surface tension titration measurements to show that both 2-hydroxyhexanoic acid (HHA) and 2-hydroxyoctanoic acid (HOA) strongly favor the protonated, neutral acid form at the air–water interface. In addition, we demonstrate that IR-RAS can be used to observe changes in protonation state for soluble organic acids at the water surface *in situ* as a function of pH, providing a direct spectroscopic probe of surface dissociation. Comparison of interfacial IR-RA spectra with bulk ATR spectra confirms that the neutral, protonated α-hydroxyacid form is favored at the interface, while also suggesting that the difference in relative surface activity between the acid and the anion likely accounts for a significant percentage of the change in “effective surface-pKa” observed by surface tension measurements.

## 2 Experimental Methods

### 2.1 Materials

2-hydroxyhexanoic acid (HHA; ≥98%), 2-hydroxyoctanoic acid (HOA; ≥98%), hexanoic acid (≥99%), sodium hydroxide (≥98%), and hydrochloric acid (37%) were obtained from Sigma-Aldrich and used without further purification. All aqueous solutions were made with Milli-Q water (18.2 MΩ).

### 2.2 Surface Tension Titrations

Surface tension measurements were obtained using a Biolin Scientific Sigma 702 Tensiometer with a platinum Wilhelmy plate. pH measurements were obtained using a Thermo Fisher Scientific Orion Star A211 pH meter. Acidic solutions were prepared by dissolving 1 mM of HHA or HOA in water with a starting solution pH of 3.60 ± 0.02 and 3.56 ± 0.05, respectively. Basic solutions were prepared by dissolving HHA or HOA in water and adjusting starting pH to ∼12 using NaOH, with a final concentration of 10 mM HHA and 1 mM HOA.

Surface tension titrations were conducted following an established approach outlined by Wellen et al.^26^ 50 mL of α-hydroxyacid solution was placed in a crystallizing dish, along with a magnetic stir bar. Solutions were allowed to equilibrate for at least 30 min prior to beginning each titration. The acidic solutions were titrated with 150 mM NaOH, and the basic solutions were titrated with 100 mM HCl. The solution was stirred for 2 min following each addition of titrant. Stirring was then stopped, and the solution was allowed to equilibrate for at least 3 min prior to taking pH and surface tension measurements. Each surface tension measurement was taken in triplicate, and a standard deviation of <0.10 mN/m between measurements was used as a proxy for equilibration. As has been previously observed,^26^ data collected in the region of the largest surface tension change required a longer equilibration time, with a maximum equilibration time of ∼20 min. To examine possible effects of concentration on the observed effective surface-p*K*_a_, 20 mM HOA was also studied, as discussed in the Supporting Information.

For surface tension titrations with acidic solutions, the bulk p*K*_a_ of HHA and HOA were determined in tandem for each trial, using the measured pH values and volume of base added. This was possible because the initial acidic solutions were composed of only HHA or HOA, and the initial pH was not adjusted by the addition of HCl, as is sometimes done for these measurements.^26^ Additional potentiometric titrations of 10 mM HHA, 50 mM HHA, and 20 mM HOA were conducted using 4.0 M NaOH as the titrant. Gran plots^67^ were used to extract bulk p*K*_a_ values from all potentiometric titrations, as discussed in the Supporting Information (Figures S1–S4).

### 2.3 Infrared Spectroscopy

For the infrared spectroscopic studies, solutions of organic acids were prepared by dissolving each species in water, adjusting the pH through addition of NaOH from a 1.0 M stock solution, and diluting to a final solution concentration of 50 mM HHA, 20 mM HOA, and 75 mM hexanoic acid. The pH of each solution was measured using a Labquest2 with a Vernier pH meter. For the α-hydroxyacids, both infrared (IR) reflection–absorption (RA) spectra and attenuated total reflectance (ATR) spectra were taken of each solution, allowing for direct comparison between the surface and bulk behavior of matched solutions.

### 2.4 Infrared Reflection–Absorption Spectroscopy

IR reflection–absorption spectra of HHA and HOA at the air–water interface were obtained using a home-built IR-RAS setup and methodology that has been previously described.^64–66^ Briefly, 50 mL of solution was placed in a PTFE Langmuir trough (KSV NIMA, 14.5 × 7 × 0.5 cm^3^), and the external MIR beam of a Bruker Invenio-R spectrometer was focused using a CaF_2_ lens. A ZnSe holographic wire grid polarizer (Thorlabs) was used to select for s-polarized light, which enhances the signal-to-noise. The beam was then reflected off the water surface using 2” gold mirrors at an angle of 60° relative to the surface normal of the water and collimated by a gold parabolic mirror before reaching the detector. The front-end of the IR-RAS setup (Langmuir trough, detector, and external optics) was enclosed and purged with CO_2_-scrubbed dry air before and during all data collection. At the 60° angle of incidence used in these experiments, absorption using s-polarized light gives a negative signal. Spectra were recorded and processed using OPUS software from Bruker from 250 individual scans and were collected at 1 cm^–1^ resolution using a zero-filling factor of 1. The spectra presented here are averaged across three spectra collected consecutively and are calculated as reflection-absorbance (RA), where RA = −log­(*R*/*R*_0_). R is the reflectivity of the sample and *R*_0_ is the reflectivity of pure water. The water and sample reflectivity were measured under the same atmospheric conditions and spectrometer parameters, and the atmospheric compensation tool in OPUS was used to mitigate possible fluctuations in conditions.

### 2.5 Attenuated Total Reflectance Spectroscopy

Following measurement of the IR-RA spectra, the solutions were collected from the Langmuir trough, and ATR spectra for each solution were taken using a commercial Agilent Technologies Cary 630 FTIR ATR instrument. Spectra were collected by averaging 64 scans at 4 cm^–1^ resolution. Water was used as the background. The ATR IR spectra were used to directly compare the bulk phase to the surface spectra measured by IR-RAS.

### 2.6 Spectroscopic Analysis

Postprocessing of all spectroscopic data was conducted using Igor Pro (Wavemetrics) software. The degree of dissociation and bulk p*K*_a_ values from the ATR spectra were calculated following the method outlined in Müller et al.^68^ Because bulk p*K*_a_ values have not been previously reported for HHA and HOA, the method was validated using hexanoic acid, which has a well-known literature p*K*_a_.^61^ A modified version of this method was used to calculate the degree of dissociation and surface-p*K*_a_ values from the IR-RA spectra, incorporating a correction for differences in the relative surface activity of the protonated acid and deprotonated anion species. A detailed description of the procedures used for spectral analysis is provided in the Supporting Information.

## 3 Results and Discussion

The bulk p*K*_a_ values of 10 mM 2-hydroxyhexanoic acid (HHA) and 1 mM 2-hydroxyoctanoic acid (HOA) were experimentally determined *via* potentiometric titration (Figures S2–S3) to be 3.9 ± 0.1 and 4.0 ± 0.1, respectively (Table 1). To the best of our knowledge, this is the first report of an experimentally determined bulk p*K*_a_ value for both species, although a calculated p*K*_a_ of 3.86 for HOA has been previously reported.^66^ Lactic acid, the simplest α-hydroxyacid, has a p*K*_a_ of 3.86.^69^ Our experimental results are in reasonable agreement with these values, suggesting that the bulk p*K*_a_ of α-hydroxyacids is not strongly affected by changes in alkyl tail length. This is expected based on previous results that have shown that neither carboxylic acids^40,61^ nor α-keto acids^64,70^ have large changes in bulk p*K*_a_ with varying tail length.

**Table 1:** Experimental p*K*_a_ Values of α-Hydroxyacids

	titration			IR spectroscopy		
	bulk p*K*_a_ ^a^	effective surface-p*K*_a_ ^a^	Δp*K*_a_ ^b^	bulk p*K*_a_ ^c^	surface-p*K*_a_ ^c^	Δp*K*_a_ ^b^
HHA	3.9 ± 0.1	4.5 ± 0.2	0.6 ± 0.2	3.78 ± 0.03	4.0 ± 0.1	0.2 ± 0.1
HOA	4.0 ± 0.1	5.41 ± 0.05	1.4 ± 0.1	4.0 ± 0.1	4.4 ± 0.2	0.4 ± 0.2

a Reported values represent an average of three independent titrations with a reported uncertainty of one standard deviation. Bulk p*K*_a_ values were obtained from potentiometric titrations of 10 mM HHA (Figure S2) and 1 mM HOA (Figure S3) and effective surface-p*K*_a_ values were obtained from surface tension titrations (Figure 2).

b Δp*K*_a_ is the difference between the surface-p*K*_a_ and the bulk p*K*_a_ with propagated uncertainty.

c Reported values represent the value from fitting a sigmoid to the average degree of dissociation obtained from spectra with a reported uncertainty of one standard deviation from the fit. Bulk p*K*_a_ values were obtained from ATR spectra (Figure S13), and surface-p*K*_a_ values were obtained from IR-RA spectra (Figure 2).

In this study, we have used the concentration of α-hydroxyacids as their activity, assuming an activity coefficient of unity for all species, which is a standard approximation for these studies.^25,26,28,51^ We examined bulk p*K*_a_ as a function of concentration *via* potentiometric titrations, as discussed in the Supporting Information. The bulk p*K*_a_ value obtained for 50 mM HHA (3.8 ± 0.1) is the same as 10 mM HHA (3.9 ± 0.1) within experimental error, as is also found for 1 mM (4.0 ± 0.1) and 20 mM HOA (3.92 ± 0.07), suggesting this is a reasonable approximation.

### 3A Determination of Effective Surface-p*K*_a_ *via* Surface Tension Titration

Surface tension titrations have been developed as a robust tool for assessing the protonation state of medium-chain organic acids at the air–water interface,^26,38,60^ and we follow this established methodology for both HHA and HOA. Briefly, 10 mM HHA and 1 mM HOA solutions were prepared with a starting pH of ∼12. These solutions were then titrated from high pH to low pH by the addition of 100 mM HCl. The solution was allowed to equilibrate after each addition of titrant, and the surface tension was measured in triplicate (Figure S5). The surface tension measurements were then fit to a surface activity model given by eq 1, and the “effective surface-pKa” was obtained by minimizing the sum of the squared deviation between experimental and fit values. As described in the introduction, the effective surface-p*K*_a_ is a useful quantitative metric of the pH-dependent distribution of protonated and deprotonated species present at the water surface, which allows for comparison between molecules. Representative data showing change in surface tension and the fitted surface activity model are given in Figure 2 for both HHA (Figure 2A) and HOA (Figure 2B), with absolute surface tension data for all trials provided in Figure S5.

**Figure 2:**
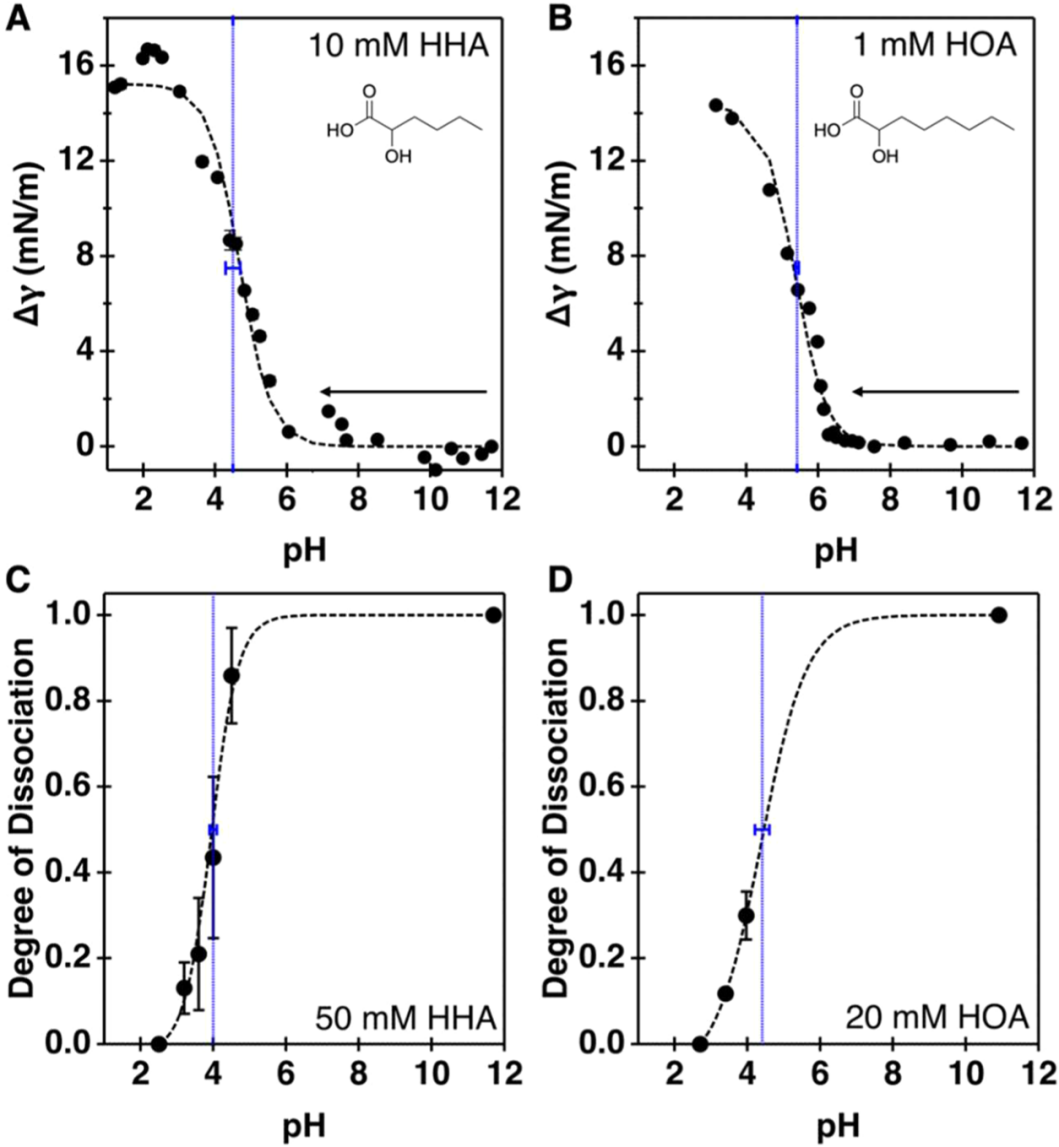
Experimental determination of the protonation state of α-hydroxyacids at the air–water interface. Panels A and B give representative surface tension data (circles) from the titration of (A) 10 mM HHA and (B) 1 mM HOA with 100 mM HCl, with an arrow showing the direction of titration. Fits given by the surface activity model are shown as dashed black lines. Surface tension data are represented as Δγ = γ_max_ – γ. Absolute surface tension values for all trials are shown in Figure S3. Error bars representing the standard deviation of triplicate surface tension measurements for each point are included but fall within the size of the markers. The average effective surface-p*K*_a_ (HHA = 4.5 ± 0.2 and HOA = 5.41 ± 0.05) is represented by the blue vertical dashed line with an error bar representing the standard deviation in calculated effective surface-p*K*_a_ across three independent trials. Panels (C and D) show the average interfacial degree of dissociation as determined by IR-RAS (circles) for 50 mM HHA (C) and 20 mM HOA (D) with a reported uncertainty of one standard deviation (see SI for details of the uncertainty calculation). Sigmoid fits are shown as dashed black lines, and the average surface-p*K*_a_ (HHA = 4.0 ± 0.1 and HOA = 4.4 ± 0.2) obtained from the sigmoid fit is represented by the blue vertical dashed line with an error bar representing the uncertainty of the fit.

The effective surface-p*K*_a_ values of HHA and HOA were determined to be 4.5 ± 0.2 and 5.41 ± 0.05, respectively (Table 1), representing the average of three independent surface tension titrations with 100 mM HCl and a reported uncertainty of one standard deviation across trials. We report effective surface-p*K*_a_ values from titration with HCl to match the standard practice in the literature but, for completeness, we also investigated the impact of the direction of titration on the effective surface-p*K*_a_, starting with an acidic solution and titrating with NaOH (Figures S7–S8). For HOA, we find an effective surface-p*K*_a_ from titration with NaOH (5.08 ± 0.05) that is lower than that obtained by titration with HCl (5.41 ± 0.05). A similar shift to lower effective surface-p*K*_a_ has been previously observed for nonanoic acid by Wellen et al.,^26^ but not by Luo et al.,^25^ suggesting the effect of direction of titration is quite sensitive to exact experimental conditions, which we discuss in the Supporting Information.

The surface tension titrations were conducted using 10 mM HHA and 1 mM HOA to allow for direct comparison with previous surface tension studies conducted at similar, low concentrations for which the surface activity model has been validated.^25,26^ However, the spectroscopic studies discussed below were conducted at higher concentrations (50 mM HHA and 20 mM HOA) because of sensitivity limitations. It has been previously observed that effective surface-p*K*_a_ can shift with concentration to either a higher or lower value. Higher values of surface-p*K*_a_ have been observed with increasing concentration of fatty acids due to “premicellar aggregation” and the formation of an acid-soap complex.^71^ In contrast, it has also been suggested that higher concentrations may lead to lower observed surface-p*K*_a_ because of a lower relative surface enrichment.^51^ To examine the effect of concentration on effective surface-p*K*_a_ for α-hydroxyacids examined here, we conducted additional surface tension titrations of 20 mM HOA with HCl (Figure S6), finding an effective surface-p*K*_a_ of 5.3. This value is slightly lower than that observed for 1 mM HOA (5.41 ± 0.05) but suggests that the differences in concentration examined here have a minimal impact on the observed results, and it is reasonable to compare the results obtained from these surface tension titrations to the spectroscopy studies.

HHA is a 6-carbon molecule with a 4-carbon alkyl chain, whereas HOA is an 8-carbon molecule with a 6-carbon alkyl tail. As observed here, HOA has a higher effective surface-p*K*_a_ than HHA. An increase in effective surface-p*K*_a_ with longer alkyl tail length is consistent with previous observations for carboxylic acids.^26,40–46^ This has been previously attributed to increased van der Waals forces between molecules with longer alkyl chains, which result in more interactions between the headgroups that make it more difficult to remove the acidic hydrogen,^26,41^ as well as to differences in the relative solvation energies of the alkyl chains between the acid and anion.^40^ Combined with the increased surface activity of longer-chained acids, this means that it is expected that the effective surface-p*K*_a_ should increase for acids with longer alkyl tails, resulting in a larger difference between the surface and bulk behavior. The relative increase in effective surface-p*K*_a_ between HHA and HOA is shown graphically in Figure 3, along with the difference between the effective surface-p*K*_a_ and bulk p*K*_a_ for both molecules.

**Figure 3:**
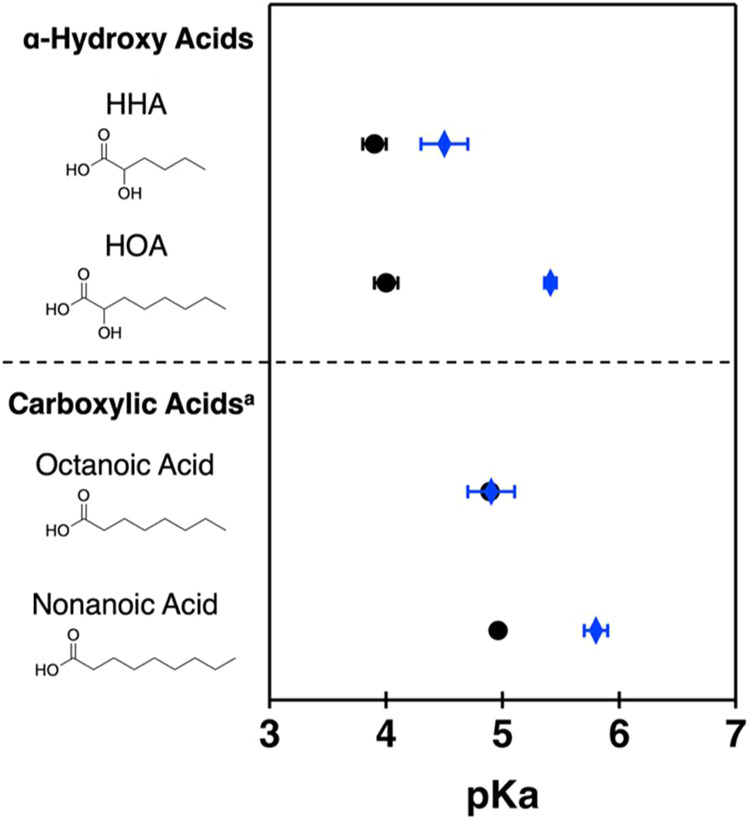
Comparison of the bulk p*K*_a_ (black circles) and effective surface-p*K*_a_ (blue diamonds) for 2-hydroxyhexanoic acid (HHA), 2-hydroxyoctanoic acid (HOA), octanoic acid, and nonanoic acid. ^a^Data for carboxylic acids is from Wellen et al.^26^

Here we observe that even HHA, which has only a 4-carbon tail, has a bulk p*K*_a_ of 3.9 ± 0.1 and an effective surface-p*K*_a_ of 4.5 ± 0.2, resulting in a Δp*K*_a_ of 0.6 ± 0.2, indicating that the protonated acid is favored at the air–water interface. HOA, with a 6-carbon tail, has a bulk p*K*_a_ of 4.0 ± 0.1 and an effective surface-p*K*_a_ of 5.41 ± 0.05, resulting in a Δp*K*_a_ of 1.4 ± 0.1. This implies that for HOA the protonated acid is significantly favored at the interface compared to the anion, and that there is a larger difference between the surface and the bulk behavior for HOA than the shorter HHA.

For both α-hydroxyacids, there is a significant increase in effective surface-p*K*_a_ compared to the bulk p*K*_a_, which can be contrasted with the previously reported behavior of fatty acids (Figure 3). Using the same surface tension titration method employed here, octanoic acid, an 8-carbon carboxylic acid, was found to have an effective surface-p*K*_a_ of 4.9 ± 0.2,^26^ which is not significantly different from its bulk p*K*_a_ of 4.89,^26,61^ giving a Δp*K*_a_ of 0 within experimental error. This is in contrast with the directly comparable α-hydroxyacid, HOA, which has a Δp*K*_a_ of 1.4 ± 0.1, despite its larger polar headgroup and shorter alkyl tail (C6 instead of C7 for octanoic acid). Nonanoic acid, the 9-carbon carboxylic acid, was found to have an effective surface-p*K*_a_ of 5.8 ± 0.1, which is significantly higher than the bulk p*K*_a_ of nonanoic acid (4.96) with a Δp*K*_a_ of ∼0.8,^26,61^ but this is still a smaller difference than that observed for HOA.

α-Hydroxyacids clearly show a larger relative shift in effective surface-p*K*_a_ compared to the bulk p*K*_a_ than is observed for fatty acids, with significant differences even for quite short-chained molecules. This difference can be understood in the context of a broad trend in observed interfacial behavior: neutral species are favored at air–water interfaces.^25–51^ α-Hydroxyacids are stronger acids than carboxylic acids. Therefore, it is reasonable that they would show a larger relative increase in effective surface-p*K*_a_. Favoring the neutral, protonated acid species at the interface requires a relatively larger change in speciation for α-hydroxyacids than is required for the weaker carboxylic acids under the same solution conditions. Although altered protonation at the interface has not been universally observed,^52–55^ there is general agreement in the literature that the interface favors neutral species.^25–51^ Regardless, our results indicate that medium-chain α-hydroxyacids favor the neutral, protonated acid form at the water surface.

### 3B Spectroscopic Determination of Interfacial Protonation State

While effective surface-p*K*_a_ is a helpful metric for comparison, it is limited in its ability to inform about protonation equilibria, as it includes contributions from the adsorption/desorption of species at the interface, in addition to changes in the dissociation equilibrium at the surface. To attempt to disentangle these processes and gain more molecular insight into the protonation state at the interface, we turn to spectroscopic techniques to directly examine differences in the protonation state of molecules *in situ* at the air–water interface compared to the bulk. Infrared reflection–absorption spectroscopy (IR-RAS) was used to obtain the vibrational spectra of both HHA and HOA at the air–water interface. For each aqueous solution, the bulk spectra were also obtained using attenuated total reflectance (ATR) IR spectroscopy, allowing for direct comparison of vibrational features between species adsorbed to the surface and solvated in the bulk. The carbonyl region of the matched ATR and IR-RA spectra are shown in Figure 4 for 50 mM HHA and Figure 5 for 20 mM HOA, across pH conditions ranging from 2.5 to 11.7. Full spectra for all solutions are given in Figures S9–S10 with detailed assignments for key spectral features given in Table S1.

**Figure 4:**
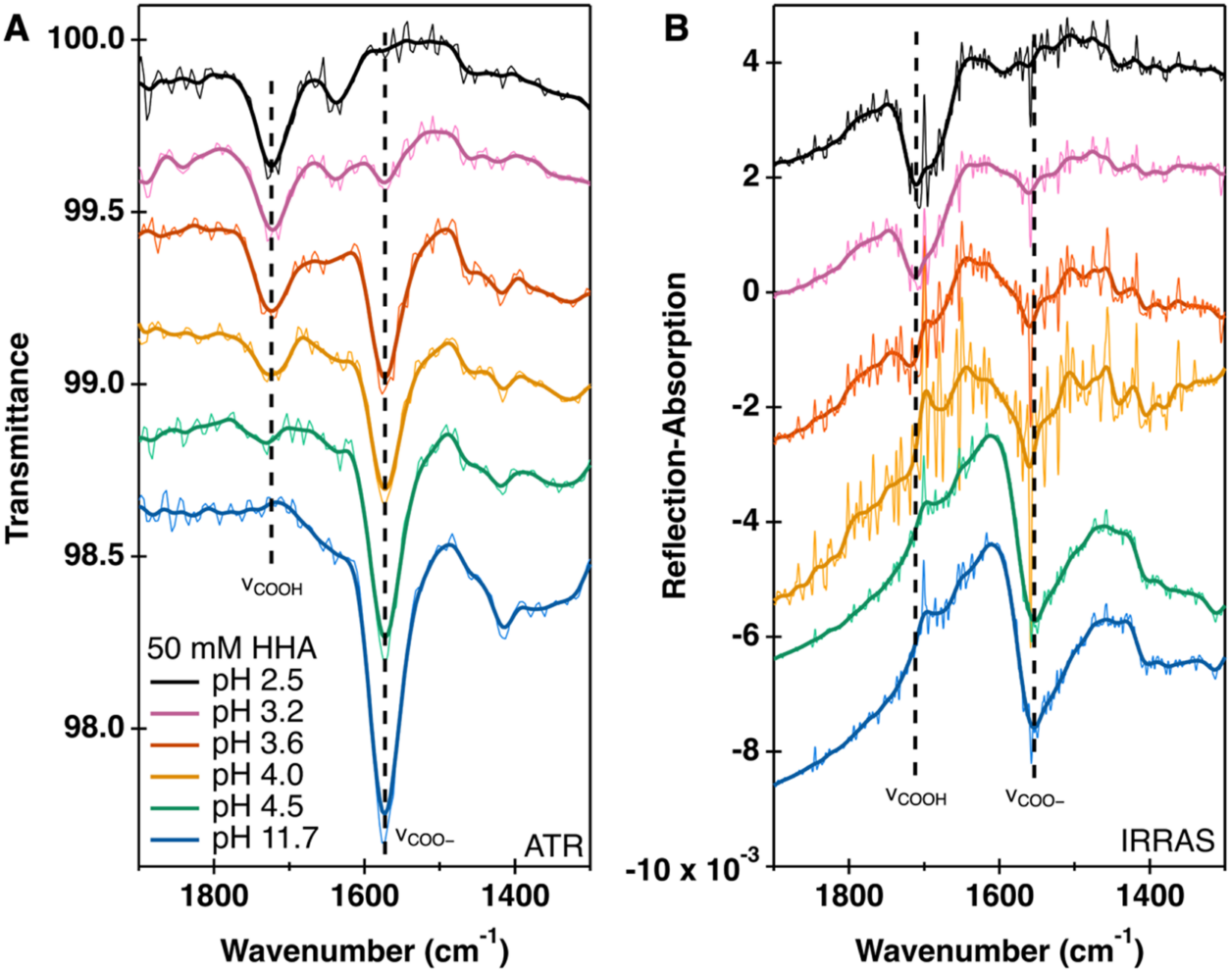
Representative ATR IR spectra (A) and IR-RA spectra (B) of 50 mM HHA at varying bulk pH (raw spectra are shown as thin lines with corresponding smoothed spectra overlaid as thick lines). The carbonyl region is shown with transitions corresponding to the carbonyl acid stretch (*ν*_COOH_ ∼ 1720 cm^–1^) and the carboxylate anion stretch (*ν*_COO–_ ∼ 1565 cm^–1^). Vertical dashed lines are drawn to guide the eye. Full spectra for all solutions are shown in Figure S9, with example fits shown in Figures S11 and S14.

**Figure 5:**
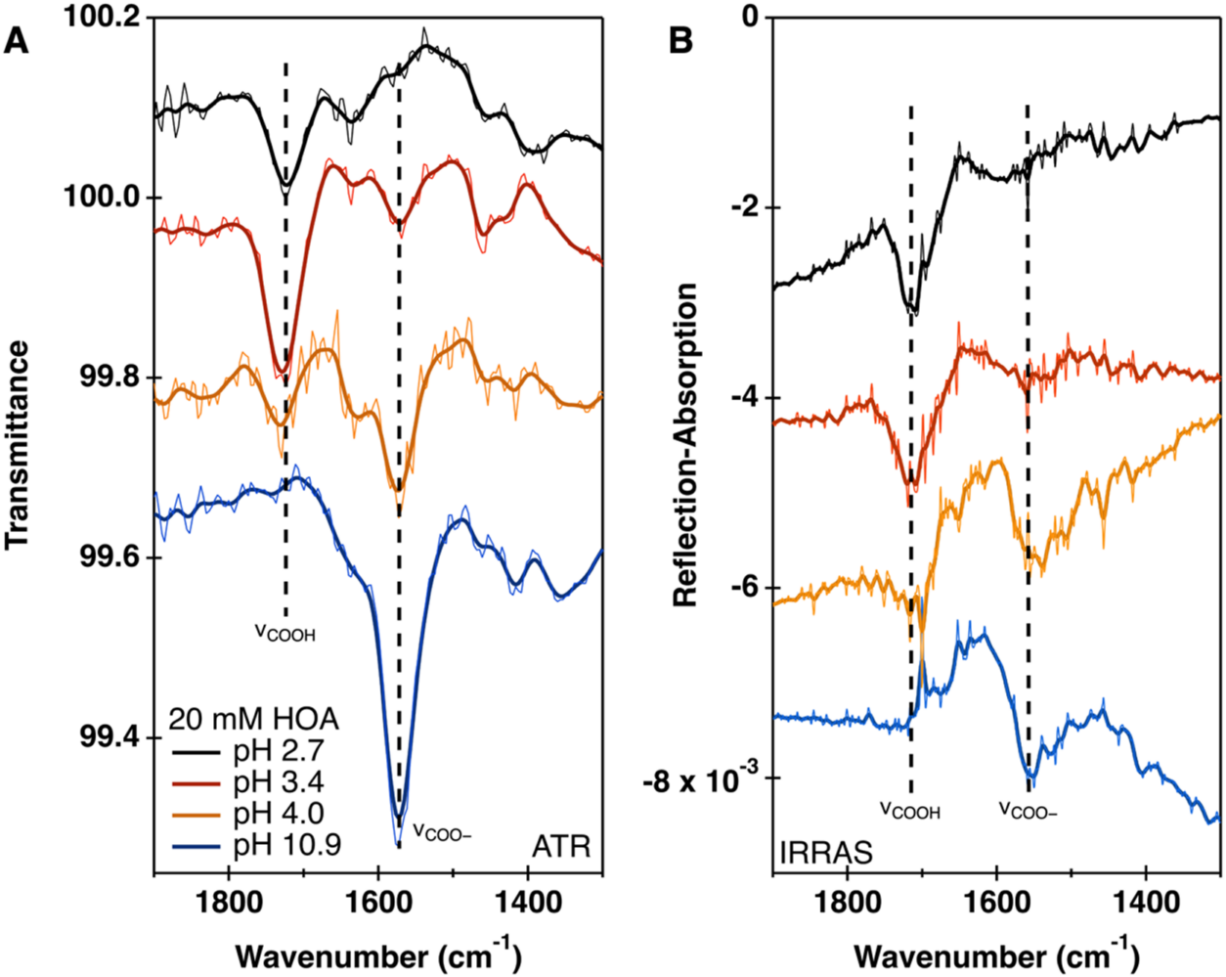
Representative ATR IR spectra (A) and IR-RA spectra (B) of 20 mM HOA at varying pH (raw spectra are shown as thin lines with corresponding smoothed spectra overlaid as thick lines). The carbonyl region is shown with transitions corresponding to the carbonyl acid stretch (*ν*_COOH_ ∼ 1720 cm^–1^) and the carboxylate anion stretch (*ν*_COO–_ ∼ 1565 cm^–1^). Vertical dashed lines are drawn to guide the eye. Full spectra for all solutions are shown in Figure S10.

The ATR spectra can be used to examine the bulk acid–base equilibrium for each solution. At low pH, the spectra are dominated by the signal at ∼1725 cm^–1^ assigned to the carbonyl stretch vibration of the carboxylic acid group (*ν*_COOH_), while at high pH the asymmetric stretch vibration of deprotonated carboxylate group (*ν*_COO–_) at ∼ 1,573 cm^–1^ is the dominant spectral feature. The IR-RA spectra, which correspond to interfacial species, show the same spectral features, but at slightly lower frequencies (*ν*_COOH_ ∼ 1,710 cm^–1^ and *ν*_COO–_ ∼ 1,555 cm^–1^). This 10–20 cm^–1^ red-shift of the carbonyl feature has been previously observed for HOA^66^ and may indicate a difference in the hydrogen bonding environment at the interface. Both the ATR and IR-RA spectra clearly show a decrease in the ν_COOH_ band with increasing pH, while at the same time the ν_COO–_ band due to the anion increases. This is expected, given the changing acid–base equilibrium as the pH of the solution is changed. However, as we will discuss in detail below, the shift in the relative ratio as a function of pH differs between the ATR and IR-RA spectra, indicating differences between the bulk and surface behavior.

We will first examine the bulk behavior. In order to quantify the observed qualitative changes, the ATR spectra for each measured pH value were fitted to two Gaussian functions (Figure S11), representing the *ν*_COOH_ and *ν*_COO–_ vibrational bands. The degree of dissociation, α, was calculated from integrated areas of each feature (*A*_COOH_ and *A*_COO–_) as shown below

2 $$\alpha =\frac{A_{\mathrm{COO}-}}{\left(A_{\mathrm{COO}-}+\left(F\right)A_{\mathrm{COOH}}\right)}$$

Where *F* is an empirical scaling factor that accounts for the larger absorption cross-section of the *ν*_COO–_ band compared to the *ν*_COOH_ band. The scaling factor, *F*, is calculated by dividing the *A*_COO–_ obtained at maximum pH, when only anion is expected to be present, by the *A*_COOH_ obtained at the minimum pH, when the acid is expected to be fully protonated. The degree of dissociation versus pH can then be fit to a sigmoid to obtain the bulk p*K*_a_ value for the solution.^68^ A detailed description of the analysis and fitting procedure used here is given in the Supporting Information, including a comparison with other potential fitting routines (e.g., Henderson–Hasselbalch).

The validity of this approach was tested using ATR spectra of 75 mM hexanoic acid (Figure S12) solutions over a pH range from ∼3–11. The experimental scaling factor, *F*, for hexanoic acid was determined to be 2.0 ± 0.3, which is similar to previously reported scaling factors for carboxylic acids.^68^ The degree of dissociation was calculated for each pH value (Table S2), yielding an experimental p*K*_a_ value for hexanoic acid of 4.80 ± 0.02, which is in reasonable agreement with the literature value of 4.88.^61^ Using the same approach for the α-hydroxyacids, scaling factors (*F*) of 2.1 ± 0.1 and 2.3 ± 0.5 were obtained for HHA and HOA, respectively. Using the average scaling factor, the degree of dissociation was calculated at each pH (Tables S3 and S4) and, using a sigmoid fit (Figure S13) the bulk p*K*_a_ values of HHA and HOA were determined to be 3.78 ± 0.03 and 4.0 ± 0.1, respectively (Table 1). These values are in reasonable agreement with the bulk p*K*_a_ values found from the potentiometric titrations above. For HOA, the same value for the bulk p*K*_a_ (within experimental error) is found by both techniques. For HHA, the bulk p*K*_a_ value found from the 50 mM ATR data (3.78 ± 0.03) is slightly lower than that obtained by potentiometric titration for 10 mM HHA (3.9 ± 0.1) but is the same within error to the bulk p*K*_a_ obtained from potentiometric titration of 50 mM HHA (3.8 ± 0.1). The slight decrease observed for the higher concentration solutions is likely due to differences in sodium concentration or ionic strength, as detailed in the Supporting Information. Because both ATR and IR-RA spectra were taken of the same solutions, a direct comparison of protonation state can be made between the bulk and the surface for each solution.

To quantify the degree of dissociation for the IR-RA spectra, we followed a similar fitting procedure to that used for the ATR spectra, fitting the integrated areas of the *ν*_COOH_ and *ν*_COO–_ vibrational bands (Figure S14). Extracting quantitative information from this region of the IR-RA spectrum is challenging because of the presence of a water bending mode at ∼1650 cm^–1^ that can cause interference in the observed signal.^65,72^ Additionally, the refractive index and reflectivity are wavelength-dependent, often causing uneven baselines in IR-RA spectra. Because of these challenges, we examined how using different baseline models and explicitly incorporating the water bending mode affected the ratio of the areas of the transitions of interest, as described in the Supporting Information. The integrated areas used in the degree of dissociation calculations reported here reflect averages across independent measurements as well as different fitting routines, with reported error of ±1 standard deviation across trials and fits, to avoid overstating the certainty of the reported quantitative values.

A qualitative comparison of the ATR (i.e., bulk) and IR-RA (i.e., interface) spectra in Figures 4 and 5 shows that the relative difference in intensity of the *ν*_COO–_ signal at high pH compared to the intensity of the *ν*_COOH_ band at low pH is not as large for the interfacial spectra as observed for the bulk. For example, in the bulk ATR spectra of HOA, at pH 10.9 the intensity of the *ν*_COO–_ band is clearly significantly larger than the intensity of the *ν*_COOH_ band at pH 2.7, due to the larger absorption cross-section of the *ν*_COO–_ band. However, for the same peaks in the IR-RA spectra, the intensity of the *ν*_COO–_ band is comparable to that of the *ν*_COOH_ band. This difference is due to the difference in the surface activity of the protonated acid and deprotonated anion species, as outlined below.

In bulk solution, a given concentration of an α-hydroxyacid will exist only in the protonated acid form at low pH and will exist only in its deprotonated anion form at high pH. Because ATR spectroscopy probes bulk solutions, the number of molecules contributing to the observed signal should be the same at both low and high pH, given that the total solution concentration is the same (e.g., 20 mM). Therefore, differences in transition intensities at high and low pH can be attributed solely to differences in the absorption cross-section of the respective transitions. At the interface, however, the situation is more complicated, as the number of species present at the water surface changes with varying pH (Figure 1).

As discussed previously, for medium-chain organic acids that act as soluble surfactants, the deprotonated anion is more soluble than the protonated acid form. This means that for solutions with the same bulk concentration, at high pH when the acid is fully deprotonated fewer anion molecules will be present at the air–water interface compared to the number of acid molecules that are present at low pH when the acid is fully protonated. Therefore, the relative intensity of the *ν*_COO–_ transition at the interface at high pH reflects absorption from fewer molecules than the intensity of the *ν*_COOH_ transition at low pH in IR-RAS. We note that while the anion is less surface active than the acid, the clear presence of the *ν*_COO–_ band in the IR-RA spectra for all but the lowest pH solutions indicates that a significant population of anion molecules is present at the interface for the bulk concentrations used in the spectroscopic studies (e.g., 20 mM for HOA).

Determining the absolute concentration of species at the interface is challenging, but it is possible to quantify the relative number of molecules at the interface across pH. We use the intensity of the α-hydroxyacid alkyl tail C–H stretches, which is assumed to be independent of protonation state, as a proxy for surface concentration. We also assume the relative orientation of the species at the interface does not change significantly as a function of pH, as detailed in the Supporting Information. To do this, we examined the relative intensity of the transitions in the C–H stretching region of the IR-RA spectrum for both HHA (Figure S15) and HOA (Figure S16) at low and high pH. In this region, we observe transitions corresponding to the CH_3_ asymmetric stretch (*ν* = 2961 cm^–1^), the CH_2_ asymmetric stretch (*ν* = 2925 cm^–1^), and CH_2_ symmetric stretch (*ν* = 2860 cm^–1^) found in the alkyl tail. By comparing the integrated areas of the C–H stretching transitions at high pH (only anion is present) to low pH (only acid is present), we can obtain the relative surface concentration of the anion compared to the protonated acid. A detailed description of this fitting procedure is given in the Supporting Information.

Using this approach, we confirm that the relative surface concentration of the anion at high pH is lower than that of the acid at low pH (Table S5). By normalizing to the surface concentration of the protonated acid form at low pH, we find that the relative surface partitioning of the anion is 68 ± 3% of that of the protonated acid for 20 mM HOA and 70 ± 10% for 50 mM HHA. The correction for surface activity was then incorporated into the degree of dissociation calculation (eq 2), as discussed in the Supporting Information, using a modified scaling factor, F*

3 $$\alpha =\frac{A_{\mathrm{COO}-}}{\left(A_{\mathrm{COO}-}+\left(F^{*}\right)A_{\mathrm{COOH}}\right)}$$

The degree of dissociation at the interface was then calculated for both HHA (Table S3) and HOA (Table S4) for all solutions, as shown in Figure 2.

The relative surface partitioning observed here is likely an upper estimate of the surface activity of the anion. Because the pH of the solutions was adjusted by the addition of NaOH, the sodium concentration is larger for the higher pH solutions (i.e., for 20 mM HOA, [Na^+^] = 0 mM at pH 2.7 and [Na^+^] = 30 mM at pH 10.9). It has been previously observed that the presence of sodium can increase the surface adsorption of anions.^25^ Given that the surface activity correction included here was calculated from the highest pH solution (and thus highest [Na^+^]), it is likely that this may slightly overestimate the relative number of anion molecules present at the interface in the intermediate pH solution, as these solutions have a lower sodium concentration. Thus, the values reported here for the degree of dissociation at the interface for HHA and HOA likely represent a lower estimate of the differences in the protonation state at the interface compared to the bulk.

In comparing the degree of dissociation calculated from the IR-RA spectra to that from the ATR spectra (Tables S3–S4), we see a clear shift to a lower degree of dissociation under intermediate pH conditions for both HHA and HOA. For HHA at pH 3.6, the degree of dissociation in the bulk is 0.37 ± 0.03, while at the surface it is 0.2 ± 0.1, a ∼45% percent difference in deprotonation between the surface and the bulk. This is also observed in the pH 4.0 HHA solution. For HOA at pH 4.0, the degree of dissociation in the bulk is 0.47 ± 0.09, while at the surface it is 0.2 ± 0.1, a ∼57% difference in deprotonation between the surface and the bulk. Significant differences in deprotonation between the bulk and surface are not observed at high and low pH conditions, which is likely due to limitations in fitting low intensity transitions with complicated baselines. It is, however, possible that differences in the protonation state at the interface are enhanced when at a pH near the bulk p*K*_a_.

The degrees of dissociation calculated from the IR-RA spectra reflect the protonation state at the interface, and by using the C–H stretches of the alkyl tail as an internal measure of surface activity, we have accounted for the differences in the surface activity between the deprotonated anion and the protonated acid. Therefore, by fitting the interfacial degree of dissociation values as a function of pH to a sigmoid (Figure 2), we are able to extract “surface-pKa” values from the IR-RAS data of 4.0 ± 0.1 for HHA and 4.4 ± 0.2 for HOA (Table 1). Unlike the effective surface-p*K*_a_ values obtained from surface tension measurements, the values obtained from the IR-RAS data have been corrected for the relative surface activity of the anion and will give a better estimate of the “true” surface dissociation equilibrium present at the interface.

For HHA, spectra taken of the same solutions give a bulk pK_a_ of 3.78 ± 0.03 *via* ATR and a surface-p*K*_a_ 4.0 ± 0.1 *via* IR-RAS, giving a Δp*K*_a_ of 0.2 ± 0.1. For HOA, the bulk pK_a_ of 4.0 ± 0.1 obtained by ATR and the surface-p*K*_a_ of 4.4 ± 0.2 found by IR-RAS yield a Δp*K*_a_ of 0.4 ± 0.2. We note that the uncertainty of the surface-p*K*_a_ value for HOA is relatively large and could be further constrained by the inclusion of additional samples at more pH conditions. However, for both HHA and HOA, we observe that the surface-p*K*_a_ is higher than the bulk pK_a_, confirming that the neutral species is favored at the interface. HOA also has a higher effective surface-p*K*_a_ than HHA, and there is a larger difference between the surface and the bulk behavior for HOA than the shorter HHA. Using our spectroscopic approach, we observe the same qualitative trends in surface-p*K*_a_ that are seen in the effective surface-p*K*_a_ values obtained by surface tension titrations.

Quantitatively, however, the Δp*K*_a_ obtained spectroscopically is smaller than that found by surface tension measurements. This indicates that the relative shift in the dissociation constant at the interface is modest, and that diffusion and adsorption processes play an important role in the speciation at the interface, likely accounting for a significant percentage of the difference in protonation state observed at the interface *via* surface tension measurements. Indeed, the Δp*K*_a_ for HHA (0.2 ± 0.1) is quite small, and, it is possible, given the relatively large experimental uncertainties in these spectroscopic studies, that this difference from the bulk p*K*_a_ may be within our experimental uncertainty. However, because we believe our approach to data analysis results in reported values that are a lower estimate of the difference in protonation state at the interface (discussed in more detail below), we suggest that HHA has a slightly higher surface-p*K*_a_ than bulk p*K*_a_. The comparatively modest changes in surface-p*K*_a_ observed for HHA and HOA using IR-RAS are reasonable, given previous literature observations that differences in surface activity between the anion and acid can account for the shift in effective surface-p*K*_a_ observed for nonanoic acid that has been determined from surface tension measurements.^25^

The presence of sodium may also account, in part, for the lower Δp*K*_a_ observed spectroscopically. As discussed previously, the presence of sodium ions stabilizes the anion species at the interface.^25^ It has also been shown that sodium can increase the relative deprotonation of organic acids.^25,73^ This suggests that under conditions with significant salt concentration, such as those found at the sea surface microlayer or on aerosol, while the neutral form of the acid is favored at the interface, it is likely that the anion form is also present and must be considered. We note that this is unlikely to account for the entire difference that is observed spectroscopically, as surface tension titration for 20 mM HOA results in an effective surface-p*K*_a_ that is similar to that observed for 1 mM HOA.

We also point out that while IR-RAS has been widely used to examine soluble surfactants,^25,26,54,64–66,74–77^ the surface specificity of these measurements is sometimes questioned,^48,74–77^ and may be species-dependent.^63,75^ Reflection–absorption spectra are composed of signal only from the region of the water surface where the complex refractive index differs from either the gas phase or bulk aqueous phase.^78,79^ However, the nominal penetration depth of IR light extends well beyond a single surface layer (∼microns),^77,78^ which allows for the possibility of contributions in the observed spectra from “sub-surface” species, corresponding to signal from multiple reflections. Signal derived from “sub-surface” species could potentially account for the comparatively lower change in surface dissociation from the bulk, if some “bulk” or “near-bulk” behavior was mixed in, where the relative shift in dissociation is likely to be lower. However, previous work on α-hydroxyacids, including both lactic acid and HOA, has shown that contributions to IR-RA spectra from bulk or “subsurface” effects are negligible for these species.^63^ This has led to the suggestion that the “information depth” of IR-RAS for these species may be closer to ∼50 Å,^79^ making IR-RAS a suitable technique for examining the interfacial behavior of soluble organic acids. Based on this prior work, we conclude that contributions from “bulk” signal are unlikely to interfere with the observations here. However, if any contribution from the bulk is present, this would, combined with our approach to data analysis, mean that the shift in surface-p*K*_a_ reported here is a conservative estimate of the difference in dissociation at the interface.

While the reported surface-p*K*_a_ from IR-RAS maybe a lower limit, there is still a clear shift at the air–water interface to favor a lower degree of dissociation for both HHA and HOA than in the bulk, even after accounting for differences in the relative surface activity of the anion and acid. The approach used here provides a new analytical method for *in situ* spectroscopic observation of protonation state at the air–water interface that can directly probe dissociation occurring at the air–water interface.

## 4 Conclusions

In this work, we have used both surface tension titrations and IR-RAS to determine the protonation state of two medium-chain α-hydroxyacids at the air–water interface. In both independent techniques, we clearly observe a shift to favor a lower degree of dissociation for both HOA and HHA at the air–water interface than in the bulk. The neutral form of these α-hydroxyacids is preferred at the interface, which is consistent with the majority of previous studies. We also observe that the relative size of the effect increases with increasing tail length (and, therefore, surface activity), as has been previously observed for fatty acids.^26^ Because α-hydroxyacids are stronger acids than fatty acids, the preference for the neutral form at the interface results in a larger relative increase in effective surface-p*K*_a_, than is observed for comparable fatty acids.

We also demonstrated a new analytical method for *in situ* spectroscopic observation of changes in protonation state at the air–water interface that is capable of directly examining surface dissociation. Vibrational sum frequency generation spectroscopy has been previously used to investigate the degree of dissociation of soluble organic acids at the interface,^28,48^ and we suggest that IR-RAS can be used as a complementary and more accessible spectroscopic method for such studies. This technique should be further optimized in the future to help reduce the relatively large experimental uncertainty in these studies. For instance, studies conducted using D_2_O in the aqueous subphase may help disentangle the interference of the water bending mode at ∼1650 cm^–1^ with the carbonyl region. Further work examining the information depth of IR-RAS would help clarify the potential contribution of subsurface species. In addition, a systematic treatment that controls for sodium concentration is needed to better understand the role of ionic strength and activity in these systems, including investigation of potential differences in activity at the interface compared to the bulk. A full treatment of bulk and interfacial activity is likely to be particularly important to fully extrapolate these results to the high ionic strength conditions found in the ocean and on aerosol.^80–86^ Nevertheless, using this new experimental approach, we observed that the change in surface dissociation equilibrium is relatively modest, and differences in the surface activity of the acid and anion species likely account for a significant percentage of observed changes in protonation state at the interface.

These studies speak to the importance of understanding, in detail, the speciation at air–water interfaces as a function of solution conditions, as bulk pH alone is not sufficient to predict the distribution of species present at the interface. The protonated form of these acids is stabilized at the interface when under pH conditions that would favor the deprotonated form in the bulk. However, under environmentally relevant conditions, especially those with significant sodium concentration, the presence of anion at the interface cannot be discounted.

These results highlight the importance of controlling for adsorption and desorption processes in any studies that examine the changes in protonation state at the interface, as the “effective surface-pKa” obtained by surface tension measurements clearly incorporates these contributions, as we and others^25,51^ have shown. It is also clear that sodium concentration and ionic strength plays an important role in the protonation state at the interface, which is still not fully understood. Fundamental studies characterizing the speciation and surface activity at interfaces add to the larger understanding of interfacial reactivity as well as how organic films mediate the optical and physical properties of atmospheric aerosols.

## Supplementary Material



## Notes

Determination of bulk p*K*_a_ of α-hydroxyacids by potentiometric titration, surface tension titration data, effect of direction of titration on effective surface-p*K*_a_, ATR spectra and tabulated degree of dissociation for hexanoic acid, ATR and IR-RAS spectra of HHA and HOA with detailed spectral assignments, description of spectroscopy analysis procedures with representative example fits, and tabulated degree of dissociation values for HHA and HOA (PDF)
