# Supplementary material for: Changes in Protonation State of Atmospherically Relevant α‑Hydroxyacids at the Air–Water Interface Measured by Surface Tension and IR-RAS
Source: J Phys Chem A. 2025 Jul 29;129(31):7170–82. doi: 10.1021/acs.jpca.5c02825 (PMC12338067; doi:10.1021/acs.jpca.5c02825)
Supplement: Supplementary file 1 [file jp5c02825_si_001.pdf]

Changes in Protonation State of Atmospherically-Relevant α-Hydroxyacids at the Air-Water Interface Measured by Surface Tension and IR-RAS

Burgess E. Rugeley†, Katherine R. Holt†, Erica B. Peterson, Selma Moulai-Khatir, Claire F. N. Koltun, Rebecca J. Rapf*

Trinity University, Department of Chemistry, San Antonio, TX, 78212
†These authors contributed equally, *corresponding author (email: rrapf@trinity.edu)

Supporting Information

S1. Determination of Bulk pKa from Potentiometric Titrations

The bulk pKa of α-hydroxyacids was measured in tandem with the surface-pKa for surface tension titrations conducted using NaOH as the titrant, as the volume of the base added was recorded along with pH and surface tension measurements for both 1 mM HHA and 1 mM HOA. Therefore, standard potentiometric titration curves were obtained for each trial, as shown in Figures S1A and S3A, for HHA and HOA, respectively. To obtain the bulk pKa, Gran plots[1] were constructed (representative plots shown in Figures S1B and S3B), using the following equation:

$$V_{base} = -\frac{1}{K_a}(V_{base} \times 10^{-pH}) + V_{equiv}$$

with selected data points immediately before the equivalence point for each trial. Using a linear fit, the pKa can be determined from the resultant slope. Additional potentiometric titrations were also conducted for 10 mM and 50 mM HHA (Figure S2), and 20 mM HOA (Figure S4), using 4.0 M NaOH as the titrant, to examine potential effects of concentration on bulk pKa. Each reported bulk pKa value is the average of three independent trials, and error bars represent ± 1 standard deviation.

The bulk pKa for 1 mM HOA was determined to be 4.0 ± 0.1. The bulk pKa obtained from titrations of 20 mM HOA is 3.92 ± 0.07, which is the same within experimental error. The sodium concentration at the end of the titration of the 1 mM solution at pH ~12 was approximately 3 mM, whereas for the 20 mM HOA, the sodium concentration was ~30 mM. For solutions of HHA, the bulk pKa was determined to be 4.00 ± 0.06 for 1 mM solutions, 3.9 ± 0.1 for 10 mM solutions, and 3.8 ± 0.1 for 50 mM solutions. The slight decrease observed with increasing concentration is likely a result of the increasing sodium concentration of the solutions,[2, 3] which are ~3 mM, ~20 mM, and ~60 mM at the end of the titration at when the solutions are at pH ~12. All titrations were conducted without the addition of a neutral sodium source, but changes of ionic strength within a titration do not appear to significantly affect the results obtained here, as the slopes obtained *via* Gran plot all remain highly linear (curvature is expected with changing ionic strength).[4] We have used an activity coefficient of 1 for all species in these calculations, as these are still relatively dilute solutions, and the correction for the difference in activity of [H+] under these solution conditions[4] for pH is within our experimental error.

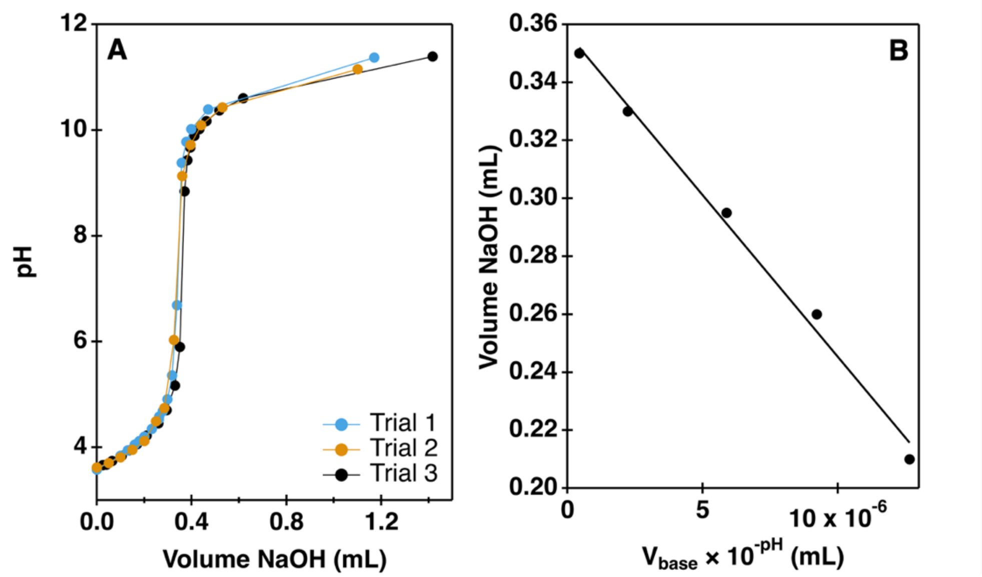

Figure S1. A) Potentiometric titrations of 1 mM HHA with 150 mM NaOH (lines connecting data points are added to guide the eye). B) A representative Gran plot of the HHA titration, using data points immediately prior to the equivalence point, with a linear fit of $y = -11162x + 0.35$, $R^2 = 0.993$. The slope can be used to determine the pKa of the solution for each trial. The bulk pKa for 1 mM HHA was determined to be 4.00 ± 0.06 from an average of three independent trials.

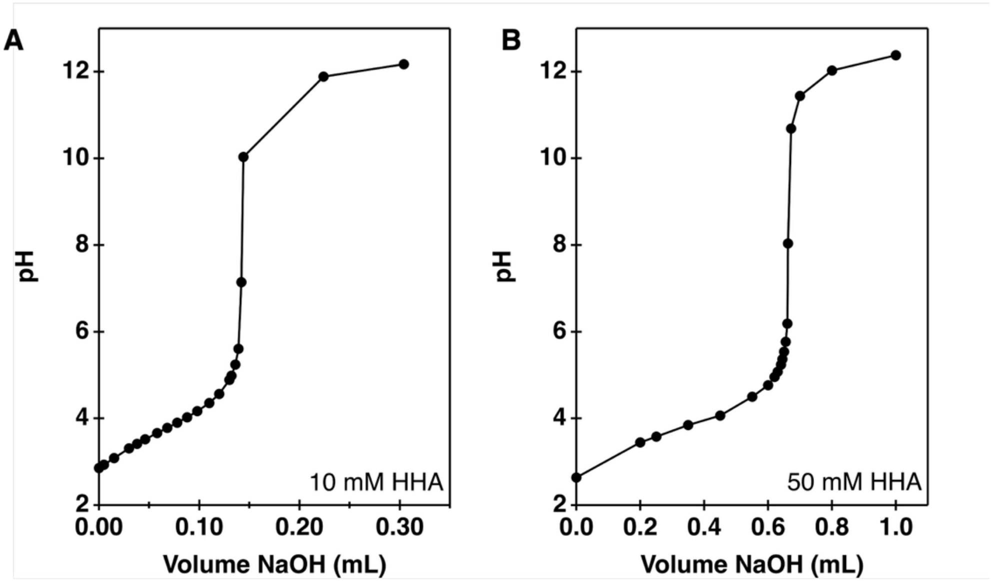

Figure S2. Representative potentiometric titrations of A) 10 mM and B) 50 mM HHA with 4.0 M NaOH (lines connecting points are added to guide the eye). Values for bulk pKa were determined using Gran plots of the data points immediately prior to the equivalence point and averaging across three independent trials. The bulk pKa values obtained for 10 mM HHA and 50 mM HHA were 3.9 ± 0.1 and 3.8 ± 0.1, respectively.

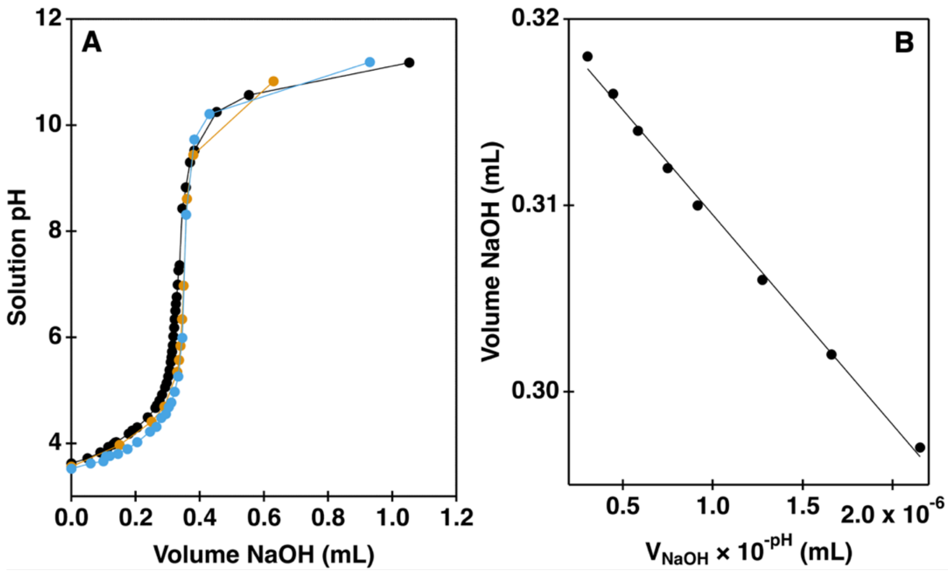

Figure S3. A) Potentiometric titrations of 1 mM HOA with 150 mM NaOH (lines connecting data points are added to guide the eye). B) Representative Gran plot of an HOA titration, using data points immediately prior to the equivalence point, with a linear fit of y = -11270x + 0.32, R^2 = 0.997. The slope can be used to determine the pKa of the solution for each trial. The bulk pKa for 1mM HOA was determined to be 4.0 ± 0.1 from an average of three independent trials.

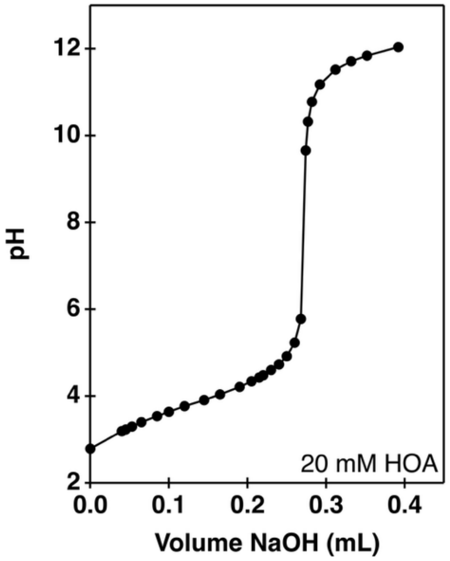

Figure S4. Representative potentiometric titration of 20 mM HOA with 4.0 M NaOH (lines connecting data points are added to guide the eye). The average bulk pKa for 20 mM HOA was determined to be 3.92 ± 0.07 across three independent trials from Gran plots of the data points immediately prior to the equivalence point.

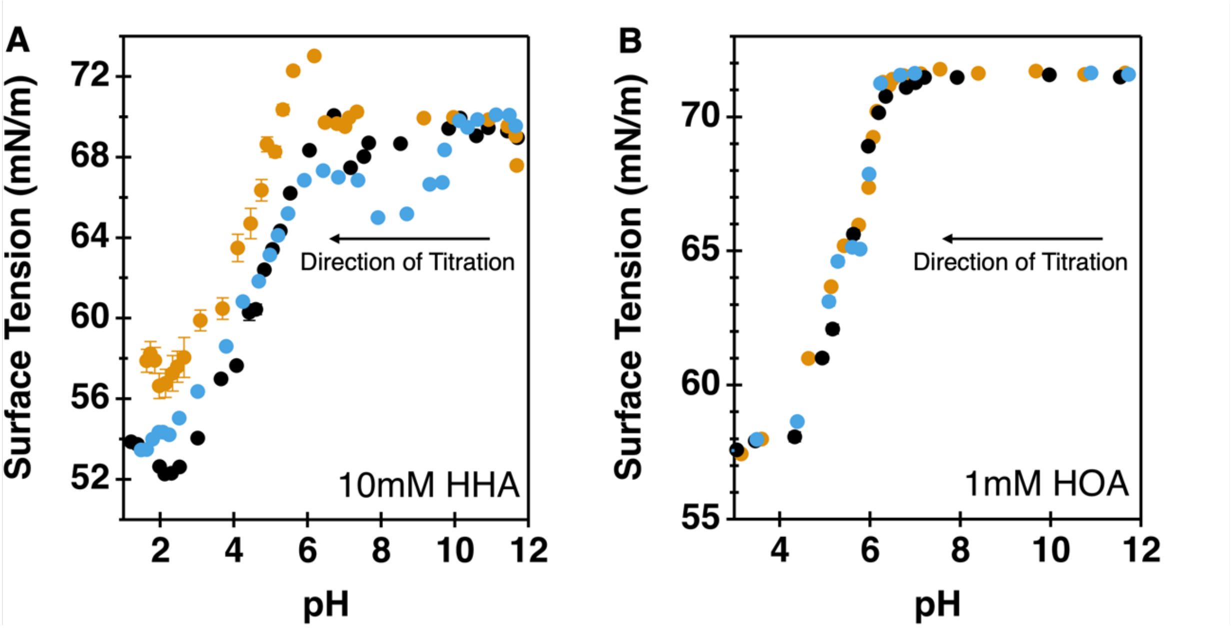

Figure S5. Surface tension versus pH titration curves for (A) 10 mM HHA and (B) 1 mM HOA conducted from high pH to low pH by addition of 100 mM HCl. The initial starting pH of samples were adjusted to ~12 by addition of NaOH. Error bars representing the standard deviation of triplicate surface tension measurements for each point are included, but most fall within the size of the markers.

S2. Effect of Concentration of Surface Tension Titration on Effective Surface-pKa

To examine the potential impact of concentration on the observed effective surface-pKa, we also conducted surface tension titrations of 20 mM HOA with HCl. The solutions were adjusted to a starting pH of ~13 with NaOH. Solutions were allowed to equilibrate in a covered, 50 mL crystallizing dish overnight and titrated with 4.0 M HCl. The solution was stirred for 2 minutes following each addition of titrant. Stirring was then stopped, and the solution was allowed to equilibrate for ~3 minutes prior to taking pH and surface tension measurements. Each surface tension measurement was taken in triplicate, and the uncertainty reported is the standard deviation between measurements. Fitting these data to the surface activity model, we obtain an effective surface-pKa of 5.3. This suggests that the differences observed between the effective surface-pKa obtained by surface tension measurements, and the surface-pKa obtained by IR-RAS are not primarily due to differences in solution concentration in the two experimental conditions.

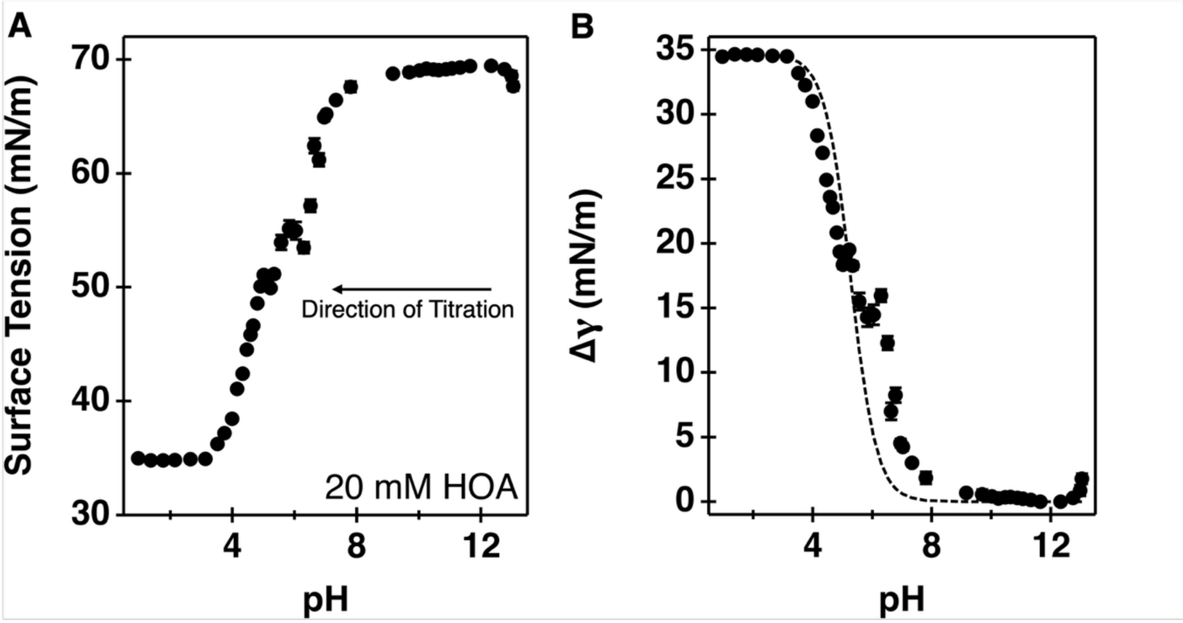

Figure S6. A) Surface tension versus pH titration curve for 20 mM HOA conducted from high to low pH by addition of 4.0 M HCl. Error bars representing the standard deviation of triplicate surface tension measurements for each point are included. B) Plot of the change in surface tension ($\Delta\gamma = \gamma_{max} - \gamma$) during the titration (circles) with the surface activity model fit (dashed black line), which gives an effective surface-pKa of 5.3 for 20 mM HOA.

S3. Effect of Direction of Surface Tension Titration on Effective Surface-pKa

In addition to conducting surface tension titrations with HCl, we also investigated the effect of the direction of titration on the resultant surface tension measurements. 1 mM HOA was titrated with 150 mM NaOH (Figure S7) from low to high pH, resulting in an effective surface-pKa of value of 5.08 ± 0.05. At the equivalence point of the titration with NaOH, the concentration of sodium is ~1 mM, whereas at the end of the titration with NaOH (pH ~12), the sodium concentration is ~3 mM. This is in contrast to the titrations with HCl, which are first adjusted to high pH by the addition of NaOH, resulting in a relatively constant sodium concentration throughout the titration of ~3-5 mM. We observe a change in effective surface-p*K*a of −0.33 ± 0.07 for HOA as a result of the direction of the titration. However, for the titration of 1 mM HHA with NaOH (Figure S8), we find an effective surface-pKa of 4.5 ± 0.1. This is the same value (within error) as was obtained from the titration of 10 mM HHA with HCl. Here, we do not observe a difference in the resultant effective surface-pKa for HHA.

A shift to lower effective surface-pKa via titration with NaOH compared to HCl has been previously observed for nonanoic acid by Wellen, et al. 2017[5]; although, interestingly because they adjust the pH of their acidic solutions to an initial pH of ~2, the titration with NaOH has a higher concentration of sodium than the titration with HCl. In contrast, Luo, et al., 2020 do not observe a difference from direction of titration for nonanoic acid when they first titrate a basic solution with HCl to low pH and then titrate the same solution back to a high pH with NaOH.[3]

These results, combined with the relatively minor change in effective surface-pKa observed for the titration of 20 mM HOA with HCl, suggest that minor differences in experimental methodology and the sodium concentration/ionic strength can have a large impact that is still not well-understood. We report these results for completeness, but these further point to the need for a systematic study of the role of sodium and ionic strength for these measurements.

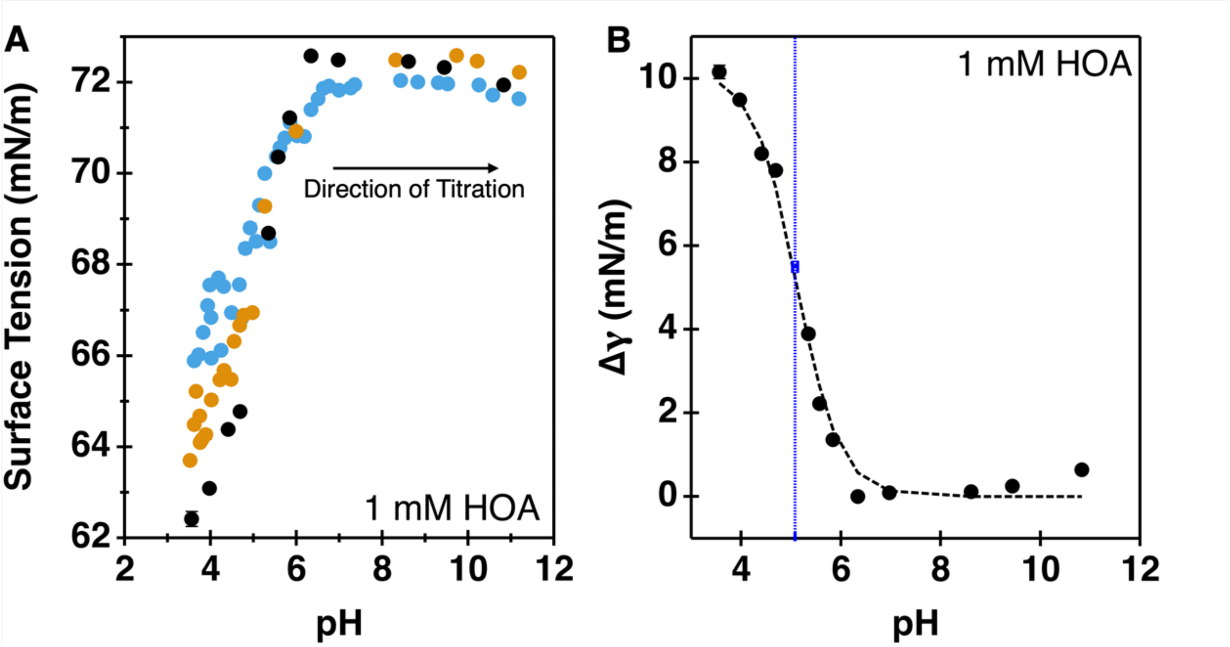

Figure S7. A) Surface tension versus pH titration curves for 1 mM HOA conducted from low to high pH by addition of 150 mM NaOH. Error bars representing the standard deviation of triplicate surface tension measurements for each point are included but fall within the size of the markers. B) Representative plot of the change in surface tension ($\Delta\gamma = \gamma_{max} - \gamma$) during the titration (circles) with the surface activity model fit (dashed black line). The average surface-pKa, 5.08 ± 0.05, is represented by the blue vertical dashed line with an error bar representing the standard deviation in calculated effective surface-pKa across three independent trials.

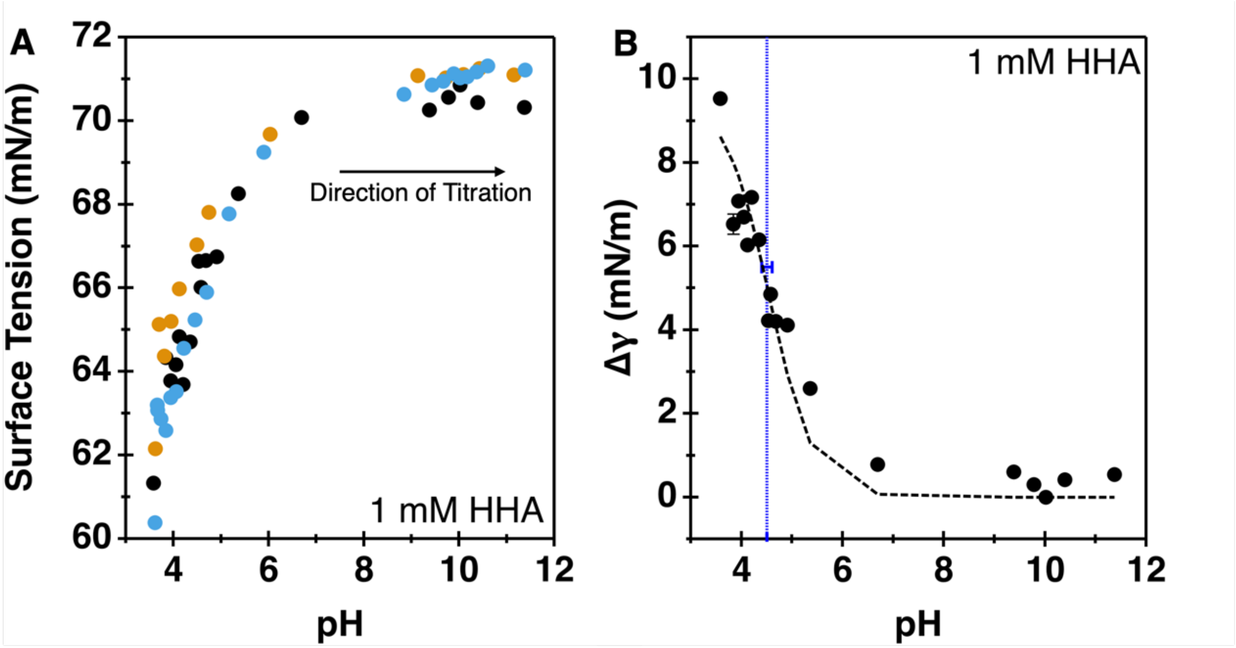

Figure S8. A) Surface tension versus pH titration curves for 1 mM HHA conducted from low to high pH by addition of 150 mM NaOH. Error bars representing the standard deviation of triplicate surface tension measurements for each point are included but fall within the size of the markers. B) Representative plot of the change in surface tension ($\Delta\gamma = \gamma_{max} - \gamma$) during the titration (circles) with the surface activity model fit (dashed black line). The average surface-pKa, 4.5 ± 0.1, is represented by the blue vertical dashed line with an error bar representing the standard deviation in calculated effective surface-pKa across three independent trials.

S4. IR Spectroscopy of α-Hydroxyacids

Table S1. Assignment of peaks for IR Spectra of HHA and HOA

	2-hydroxyhexanoic acid (HHA)		2-hydroxyoctanoic acid (HOA)	
Assignment	Aq. ATR bulk	IR-RAS, *s* surface	Aq. ATR bulk	IR-RAS, *s* surface
ν_{COOH}	1725	1710	1724	1715
ν_{COO-}	1573	1555	1573	1555
$\nu_{CH3, symmetric}$	2960	2961	Not Resolved	2961
$\nu_{CH2, symmetric}$	2930	2925	2926	2925
$\nu_{CH2, asymmetric}$	2865	2861	2859	2859

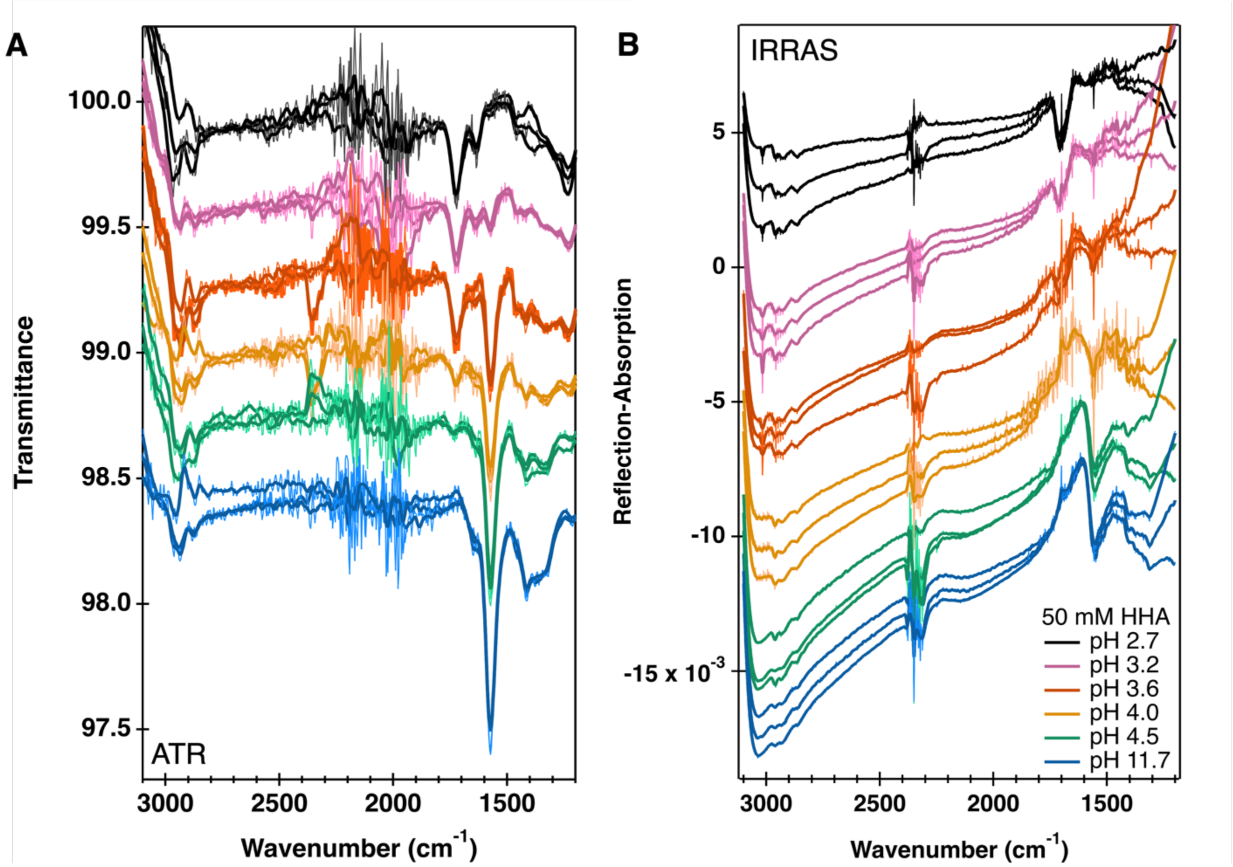

Figure S9. Full ATR (A) and IR-RA (B) spectra of 50 mM HHA as a function of pH. Raw spectra are shown as thin lines with corresponding smoothed spectra overlaid as thick lines. Spectra of the same color reflect multiple independent measurements at a given pH value.

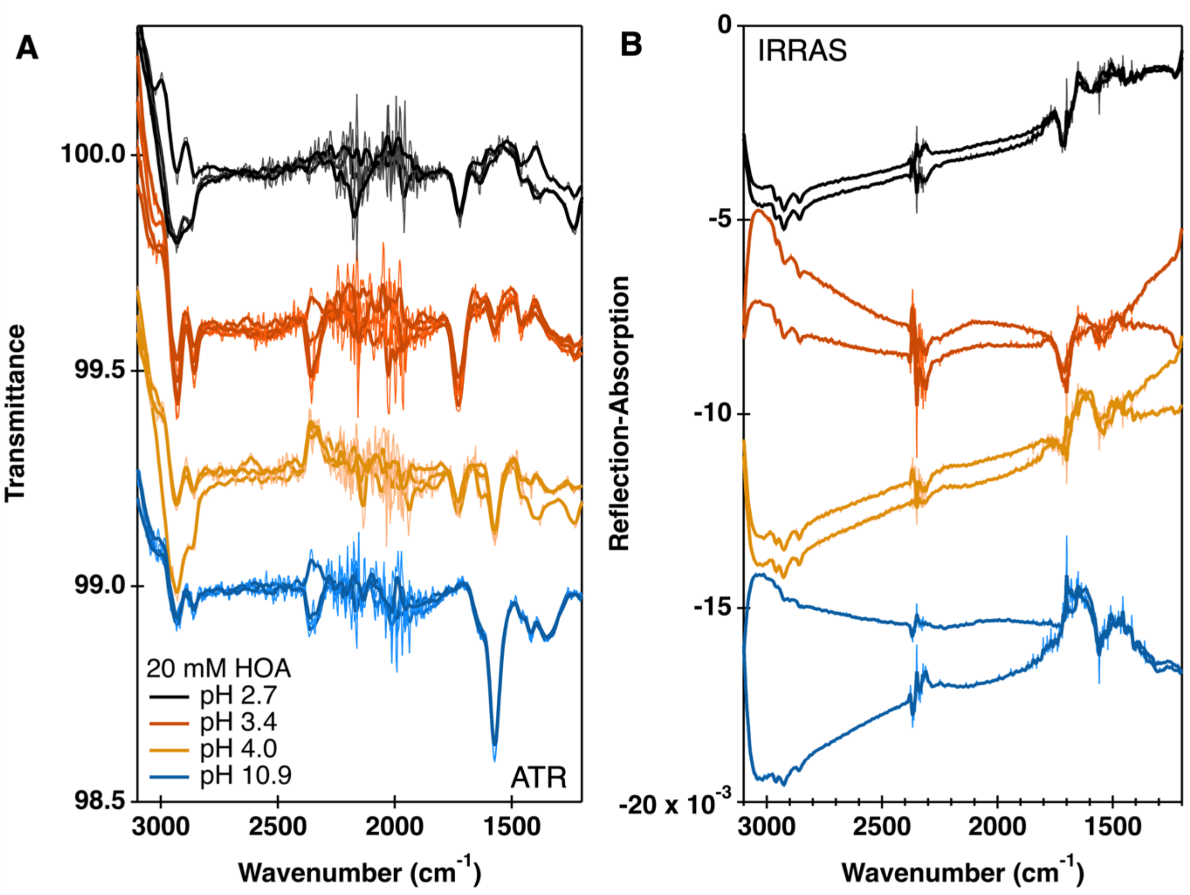

Figure S10. Full ATR (A) and IR-RA (B) spectra of 20 mM HOA as a function of pH. Raw spectra are shown as thin lines with corresponding smoothed spectra overlaid as thick lines. Spectra of the same color reflect multiple independent measurements at a given pH value.

S5. Determination of Bulk pKa from ATR Spectra

To calculate the degree of dissociation (α) from ATR spectra (Figure 3, 4, and S12), we followed the approach used by Müller, et al.[6] Because the bulk pKa of α-hydroxyacids has not been previously experimentally reported in the literature, we used 75 mM hexanoic acid to validate this analysis method, as its bulk pKa is well-established.[7]

We calculated α by using the integrated areas of the carbonyl stretch of the carboxyl (COOH) group of the acid (A_{COOH}) and the asymmetric carboxylate stretch (COO^-) of the anion (A_{COO-}), as shown below:

$$\alpha = \frac{A_{COO-}}{(A_{COO-} + (F)A_{COOH})}$$

where F is an empirical scaling factor that accounts for the differences in absorption cross-section of the ν_{COOH} band (A_{COOH}) and the ν_{COO-} band (A_{COO-}). The scaling factor, F, was calculated by dividing (A_{COO-}) obtained at maximum pH (e.g., pH 11.3 for hexanoic acid) by A_{COOH} obtained at the minimum pH (e.g., pH 3.1 for hexanoic acid), which assumes that at high pH only the anion form is present and *vice versa* at low pH. For all ATR spectra, integrated areas were obtained from the raw spectra (no smoothing), using the multi-peak fitting tool in Igor with a constant baseline

and a simple two-peak Gaussian fit between ~1480-1900 cm^{-1}. Representative examples of the fits obtained are shown in Figure S11 for 50 mM HHA at pH 3.6, pH 4.0, and pH 4.5. These are the same spectra as those shown in Figure 4. We also tested fitting procedures that incorporated a wider region, which included the symmetric carboxylate stretch ($\nu_{s,COO-}$ ~1407 cm^{-1}) (observed only for hexanoic acid), as well as explicitly including the water bending mode at ~1650 cm^{-1}, but these did not significantly change the obtained degree of dissociation, and we chose to use the simpler fitting procedure.

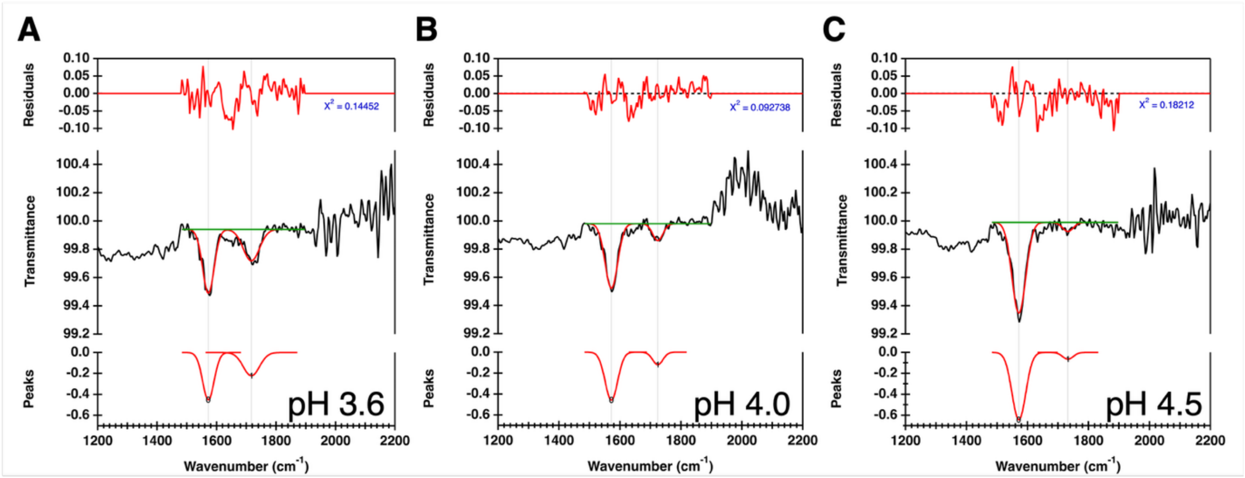

Figure S11. Representative ATR spectra (black) of 50 mM HHA at pH 3.6 (A), pH 4.0 (B), and pH 4.5 (C), showing a simple two Gaussian fit (red) with constant baseline (green) obtained from Igor multi-peak curve fitting. The top panel shows the residuals of the fit, and the bottom panel shows the fitted peaks. The fitted spectra shown here are the same spectra shown in Figure 4.

Figure S12A shows the carbonyl region of the ATR spectra of 75 mM hexanoic acid at varying pH with transitions corresponding to the carbonyl acid stretch (ν_{COOH} ~1705 cm^{-1}), the asymmetric carboxylate anion stretch ($\nu_{a,COO-}$ ~1540 cm^{-1}), and the symmetric carboxylate anion stretch ($\nu_{s,COO-}$ ~1407 cm^{-1}).[8-10] For hexanoic acid, the scaling factor, F, was determined to be 2.0 ± 0.3, by taking the ratio of the average A_{COO-} at high pH to the average A_{COOH} at low pH, obtained from three independent measurements for each pH. This value is in line with previously obtained scaling factors for similar organic acids, including propionic acid (F = 2.99 ± 0.25).[6] The degree of dissociation was then calculated for each pH (Table S2 and Figure S12B), using the average F-value. The reported degrees of dissociation represent an average of individually calculated values from 3-5 independent measurements with an uncertainty of ±1 standard deviation. Using the curve fitting tool in Igor, the average degree of dissociation as a function of pH was fit to a sigmoid (Figure S12B), including the uncertainty for each degree of dissociation as a weighting parameter. For data points where there was no experimentally determined error in the degree of dissociation (e.g. at high pH where the degree of dissociation = 1), an uncertainty of ± 0.01 was used in the fit. A detailed discussion of the choice to use a sigmoid fit to extract pKa values from the experimentally obtained degrees of dissociation is included in Section S6, including comparison to fitting using a modified Henderson-Hasselbalch equation. From this sigmoid fit, we obtain a bulk pKa of hexanoic acid of 4.80 ± 0.02, which is in reasonable agreement with the literature pKa

value of 4.88,[7] confirming the utility of this method.

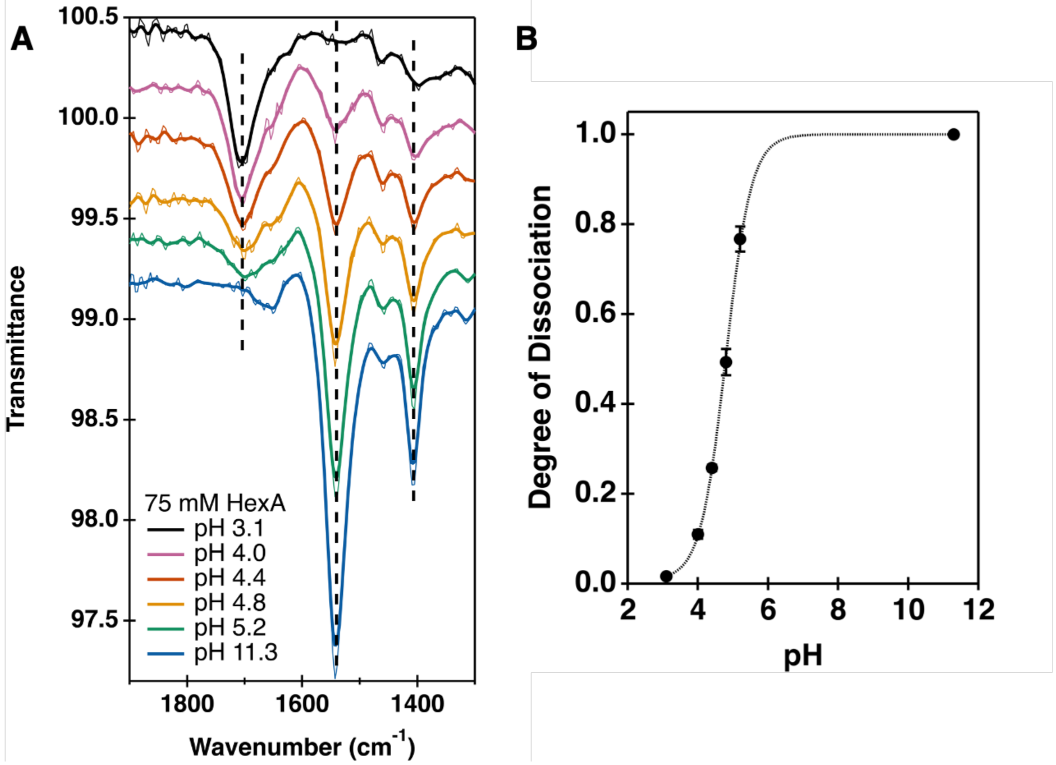

Figure S12. A) Representative ATR IR spectra of 75 mM hexanoic acid at varying pH (unsmoothed spectra are shown as thin lines with the corresponding smoothed spectra overlaid as thick lines). The carbonyl region is shown with transitions corresponding to the carbonyl acid stretch (ν_{COOH} ~1705 cm^{-1}), the asymmetric carboxylate anion stretch ($\nu_{a,COO-}$ ~1540 cm^{-1}), and symmetric carboxylate anion stretch ($\nu_{s,COO-}$ ~1407 cm^{-1}), vertical dashed lines are drawn to guide the eye. B) Plot of the degree of dissociation at each pH obtained from the ATR spectra of hexanoic acid (circles) with uncertainty of ± 1 standard deviation from at least three independent measurements. The corresponding sigmoid fit to the data (dashed line), yields a bulk pKa value of 4.80 ± 0.02.

Table S2. Degree of Dissociation for Hexanoic Acid from ATR Spectra

pH	Experimental α, using F = 2.0[a]	Expected α from literature pKa = 4.88[7]
3.1	0.017 ± 0.005	0.016
4.0	0.11 ± 0.01	0.12
4.4	0.258 ± 0.007	0.25
4.8	0.49 ± 0.03	0.45
5.2	0.78 ± 0.03	0.68
11.3	1[b]	1.00

[a]F is a scaling factor that accounts for the differences in absorption cross-section between ν_{COOH} and ν_{COO-},
[b]Signal/Noise of ν_{COOH} was too low to integrate signal, α = 1 represents fully deprotonated system

Using the same approach for the α-hydroxyacids, scaling factors (F) of 2.1 ± 0.1 and 2.3 ± 0.5 were obtained for HHA and HOA, respectively. Using the average scaling factor, the average degree of dissociation was calculated at each pH (Table S3 and S4 and Figure S13) and, using a sigmoid fit (Figure S13), the bulk pKa values of HHA and HOA were determined to be 3.78 ± 0.03 and 4.0 ± 0.1, respectively.

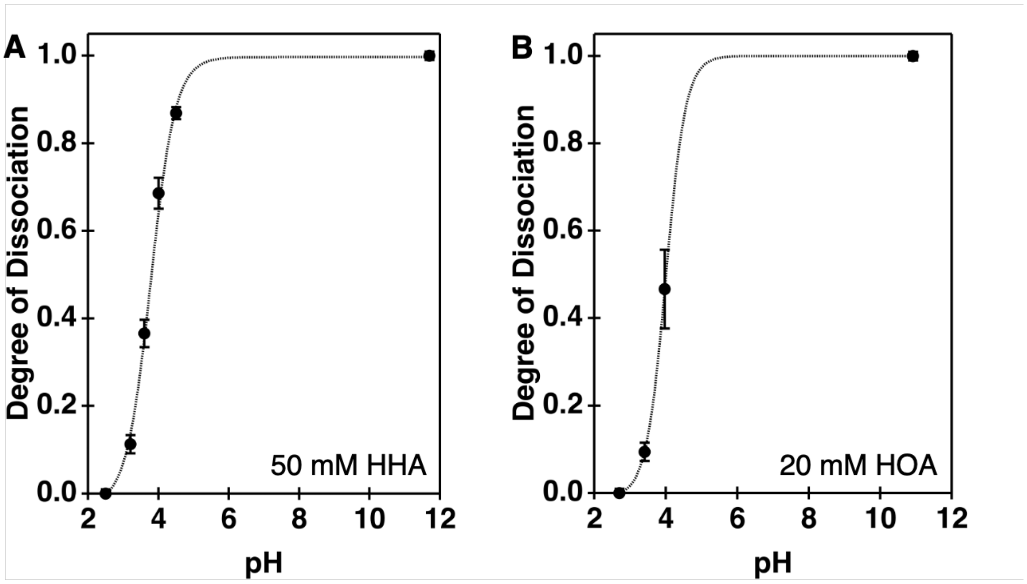

Figure S13. Plot of the degree of dissociation at each pH obtained from the ATR spectra of 50 mM HHA (A) and 20 mM HOA (B), error bars represent ± 1 standard deviation from at least three independent measurements. The corresponding sigmoid fit to the data (dashed line) yields a bulk pKa value of 3.78 ± 0.03 for HHA and 4.0 ± 0.1 for HOA.

Table S3. Comparison of the Bulk and Surface Degree of Dissociation for HHA as Determined from ATR and IRRA Spectra

pH	Bulk α, using F = 2.1[a]	Surface α, using F* = 3.0[a]
2.5	0[b]	0[b]
3.2	0.11 ± 0.02	0.13 ± 0.06
3.6	0.37 ± 0.03	0.2 ± 0.1
4	0.69 ± 0.04	0.4 ± 0.2
4.5	0.87 ± 0.01	0.9 ± 0.1
11.7	1[c]	1[c]

[a]*F is an empirical scaling factor that accounts for the differences in absorption cross-section between ν_{COOH} and ν_{COO-} for ATR, F^* is a modified scaling factor that accounts for both differences in absorption cross-section and the differences in surface activity of the acid and anion species for IR-RAS*

[b]*Signal/Noise of ν_{COO-} was too low to integrate signal, α = 0 represents fully protonated system*

[c]*Signal/Noise of ν_{COOH} was too low to integrate signal, α = 1 represents fully deprotonated system*

Table S4. Comparison of the Bulk and Surface Degree of Dissociation for HOA as Determined from ATR and IRRA Spectra

pH	Bulk α, using F = 2.3[a]	Surface α, using F = 3.4[a]
2.8	0[b]	0[b]
3.4	0.09 ± 0.02	0.10 ± 0.03
4.0	0.47 ± 0.09	0.2 ± 0.1
11.0	1[c]	1[c]

[a]*F is an empirical scaling factor that accounts for the differences in absorption cross-section between ν_{COOH} and ν_{COO-} for ATR, F^* is a modified scaling factor that accounts for both differences in absorption cross-section and the differences in surface activity of the acid and anion species for IR-RAS*
[b]*Signal/Noise of ν_{COO-} was too low to integrate signal, α = 0 represents fully protonated system*
[c]*Signal/Noise of ν_{COOH} was too low to integrate signal, α = 1 represents fully deprotonated system*

The values found from ATR spectroscopy are in reasonable agreement with the bulk pKa values found from the potentiometric titrations above. For HOA, the same value for the bulk pKa (within experimental error) is found by both techniques. For HHA, the bulk pKa value found from ATR data (3.78 ± 0.03) is slightly lower than that obtained by potentiometric titration (4.00 ± 0.06). For the potentiometric titrations used to find bulk pKa, an acidic solution of 1 mM HHA was titrated with 150 mM NaOH, resulting in a concentration of Na^+ of ~4 mM at the end of titration. In comparison, the 50 mM HHA solution analyzed spectroscopically at pH 11.7 had a Na^+ concentration of 60 mM. The presence of Na^+ ions in solution has been shown to enhance deprotonation of carboxylic acids,[2, 3] and similar differences in observed bulk pKa values have been previously attributed to differences in salinity.[3] We note that for the 20 mM HOA, the highest Na^+ concentration used was 30 mM for the ATR data, while for the titration of 1 mM HOA, the ending Na^+ concentration was ~5 mM, which is a smaller relative difference. For the 75 mM hexanoic acid measurements, the highest Na^+ concentration was 80 mM, which may account for the small difference in the experimental pKa obtained here (4.80 ± 0.02) and the literature value of 4.88.[7]

S6. Determination of Degree of Dissociation using IR-RAS

In order to fit the IR-RAS data, we used a similar fitting procedure to that used for the ATR data, with the same approach of using the integrated areas of the carbonyl stretch of the carboxyl (COOH) group of the acid (A_{COOH}) and the asymmetric carboxylate stretch (COO^-) of the anion (A_{COO-}), as shown below:

$$\alpha = \frac{A_{COO-}}{(A_{COO-} + (F^*)A_{COOH})}$$

Using F* as a modified empirical scaling factor, adapted from the corresponding ATR F-values as described below.

Extracting quantitative data from IR-RAS spectra is more challenging than ATR spectra, for several reasons, including relatively noisy spectra, baselines that are not constant, possible interference from the water bending mode at ~1650 cm^{-1} in the carbonyl region, as well as differences in the surface activity of species across a pH range. To help reduce the effects of noise, for all quantitative analysis of IR-RAS data, we used smoothed spectra (Igor, Binomial, 400).

IR-RAS spectra are calculated as reflection-absorbance (RA), where RA = $-\log(R/R_0)$. R is the reflectivity of the sample and R_0 is the reflectivity of pure water. For s-polarized light at the 60° angle of incidence used here, absorption gives a negative signal. Because the presence of organics at the surface in the sample can displace water from the interface, the water bending mode at ~1650 cm^{-1} appears in the sample spectrum as a positive feature.[11, 12] In addition to this potential interference with the carbonyl region of the organic acids, the baselines of IR-RAS data are often not flat over a given region of interest because the refractive index and reflectivity are wavelength-dependent, as can be seen in Figures S9 and S10 (as well as Figures 3, 4, S14-S16).

To account for these challenges, we examined how using different baseline models and explicitly incorporating the water bending mode affected the ratio of the areas of the transitions of interest. All data was fit using the Multi-peak Fitting Tool in Igor, using the in-built baseline models. Each feature was fitted as a Gaussian using constraints to ensure reasonable behavior, including constraining whether features were negative or positive, as well as the peak position (~20 cm^{-1} around center of peak) and width of each feature (10-40 cm^{-1}). For the carbonyl region (~1430-1830 cm^{-1}) a simple two Gaussian fit was used, except in the cases where the water bending mode at ~1650 cm^{-1} was explicitly incorporated. We fit all data to the following baseline models: cubic, log cubic, logpoly5, both with and without a feature at 1650 cm^{-1} (constrained between 1640-1660 cm^{-1}). Figure S14 shows examples of the fits obtained for a representative IR-RAS spectrum 50 mM HHA at pH 3.6. Each spectrum was fit to all 6 fitting procedures (using the 3 baseline models both with and without explicitly incorporating the water bend). The degree of dissociation was calculated from the areas obtained by each fit. Regardless of fitting procedure, an increase in the calculated degree of dissociation was observed with increasing pH. In order to not overstate the certainty of the quantitative results presented here, we have chosen to report values for degree of dissociation that represent an average across independent measurements, as well as across fitting procedures, with the reported uncertainty representing the standard deviation of the calculated degree of dissociation values across all measurements and fits for a given pH condition.

To account for the changes in surface activity, the intensity of the signal in the C-H stretching region (Figures S15 and S16) at high pH (assuming only anion is present) compared to low pH (assuming only acid is present) was used as a proxy for the relative surface concentration of the anion compared to the protonated acid. Previous studies have used the intensity of the C-H stretching transitions in IR-RA spectra to qualitatively describe the relative surface concentration of species,[3, 5, 13] including for varying concentrations of HOA (1-20 mM), using both s- and p-polarized spectra.[13]

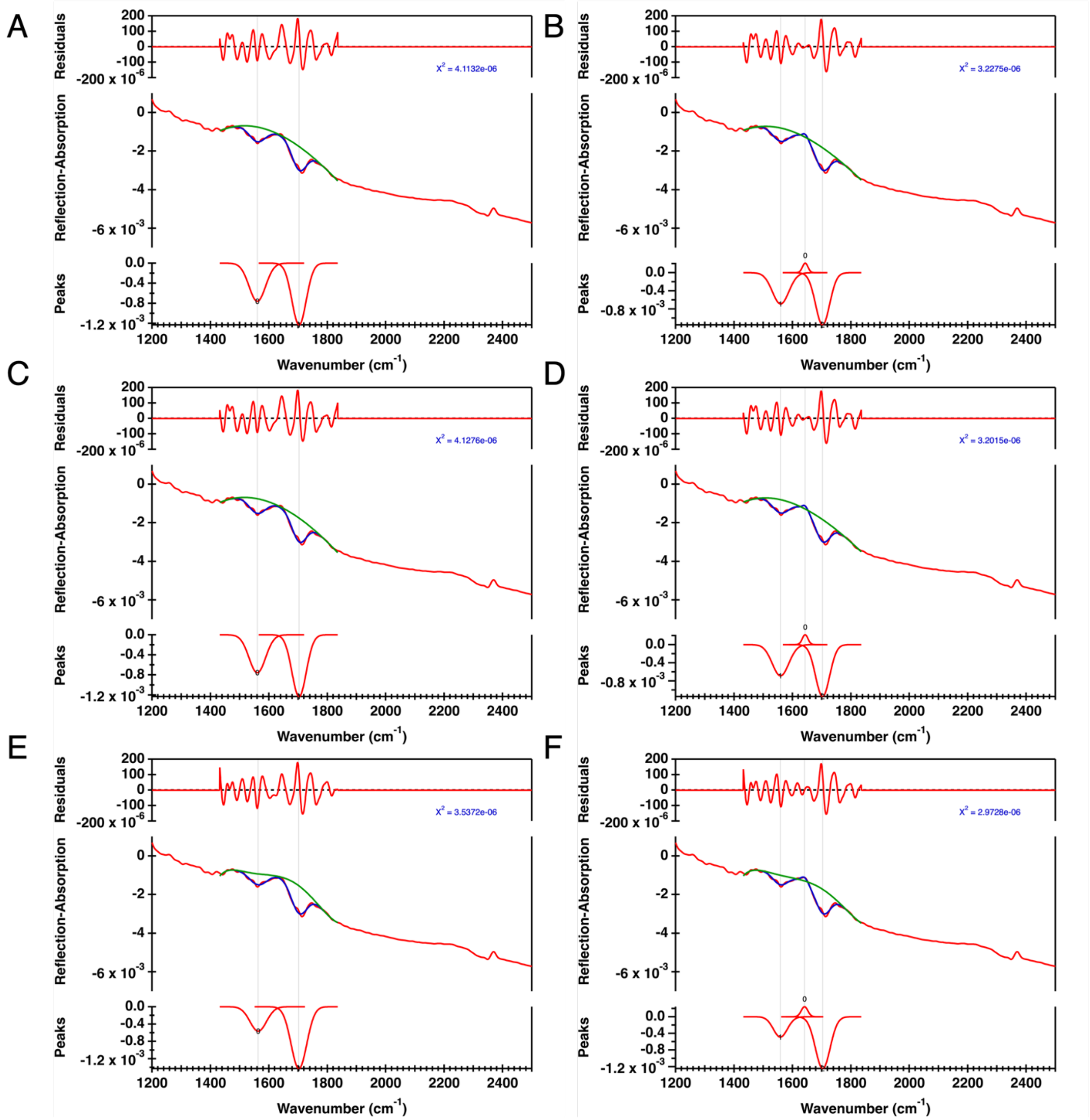

Figure S14. Representative smoothed IR-RAS spectrum of 50 mM HHA at pH 3.6 (red), showing the various Gaussian fits (blue) with obtained from Igor multi-peak curve fitting, with different baselines (green) used in the fitting parameters. A and B have a cubic baseline, C and D have a logcubic baseline, and E and F have a logpoly5 baseline. B, D, and F also include explicit fitting of a positive water feature at ~1650 cm^{-1}. The top panels show the residuals of the fit, and the bottom panels show the fitted peaks.

To quantify the relative surface activity of the acid and anion, we make a few assumptions. First, we assume that cross-sections of the C-H stretches are constant across pH conditions. We also assume that the orientation of the molecules at the interface does not change significantly as a function of pH. The s-polarized spectra collected in this study explicitly probe molecules with a

dipole that is parallel to the water surface,[11, 13] so changes in molecular orientation could also account for some differences in intensity observed in the different pH conditions. A previous study by Deal and Vaida, 2022,[13] examined HOA as a function of concentration (1 mM – 20 mM), collecting both s- and p-polarized spectra. Qualitatively, a similar decrease in C-H stretch intensity is observed with decreasing concentration in both polarization modes. This study also examined the relative change in surface ordering as a function of concentration by following shifts in the peak maximum of the asymmetric methylene stretch. While they observed some changes in ordering as a function of concentration, the relative size of this effect was small compared to differences in ordering observed for longer, insoluble surfactants, such as hydroxystearic acid.[13] In our study, we do not observe a significant shift in the transition frequency of the asymmetric methylene stretch for either HHA or HOA, which suggests that the relative change in surface ordering/molecular orientation is small across pH conditions. We believe that these assumptions are reasonable but acknowledge that they increase the relative uncertainty of this calculation.

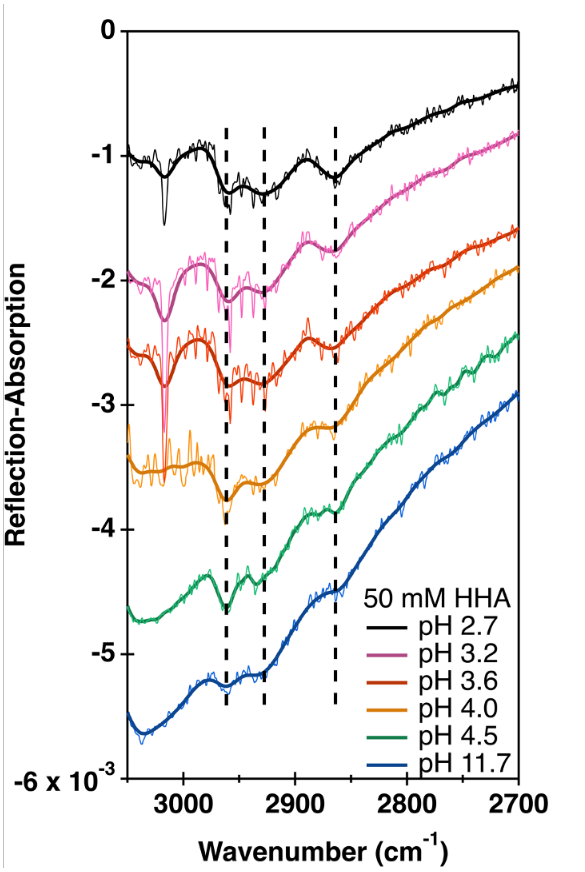

Figure S15. *Representative IR-RA spectra of 50 mM HHA at varying pH (raw spectra are shown as thin lines with corresponding smoothed spectra overlaid as thick lines). The C-H region is shown with transitions corresponding to the CH_3 asymmetric stretch (ν = 2961 cm^{-1}), the CH_2 asymmetric stretch (ν = 2925 cm^{-1}), and the CH_2 symmetric stretch (ν = 2861 cm^{-1}), vertical dashed lines are drawn to guide the eye.*

Using the Multipeak fitting tool in Igor, the integrated areas of the transitions corresponding to the CH_3 asymmetric stretch (ν = 2961 cm^{-1}), the CH_2 asymmetric stretch (ν =

2925 cm^{-1}), and CH_2 symmetric stretch (ν = 2860 cm^{-1}) were found at both high and low pH, using cubic, logcubic, and logpoly5 baseline models. All features were fitted with Gaussians and constrained to the corresponding peak position and a fixed width (~20 cm^{-1}). To account for differences in background, the average area for each transition was found for independent measurements at both high and low pH for each baseline model. The ratio of these average areas was then obtained for each baseline model for each of the C-H stretches (as shown in Table S5 for HOA). These ratios were then averaged across fitting models and all three C-H transitions to obtain the relative surface partitioning of the anion of 0.68 ± 0.03 of that of the protonated acid for 20 mM HOA and 0.7 ± 0.1 for 50 mM HHA, with a reported uncertainty of ± 1 standard deviation across measurements, fits, and transitions.

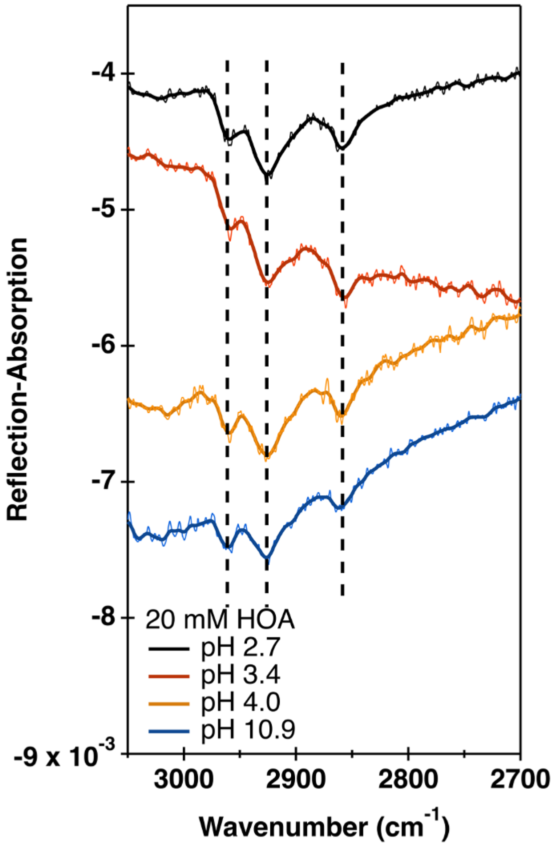

Figure S16. *Representative IR-RA spectra of 20 mM HOA at varying pH (raw spectra are shown as thin lines with corresponding smoothed spectra overlaid as thick lines). The C-H region is shown with transitions corresponding to the CH_3 asymmetric stretch (ν = 2961 cm^{-1}), the CH_2 asymmetric stretch (ν = 2925 cm^{-1}), and CH_2 symmetric stretch (ν = 2859 cm^{-1}), vertical dashed lines are drawn to guide the eye.*

We used these relative surface activities to modify the scaling parameter used in the degree of dissociation calculation. Using the scaling factor, F, found from the bulk ATR spectra, we divided by the average relative surface partitioning value found from the C-H region, yielding a modified scaling factor, F*, of 3.0 ± 0.4 for HHA and 3.4 ± 0.7 for HOA. This modified scaling factor accounts for differences in absorption cross-section, as well as the differing number of anion

versus acid species present at the interface. The appropriate F* value was then used for all calculations of degree of dissociation. We note that, while it is possible that the absorption cross-sections of the ν_{COOH} and ν_{COO^-} bands may differ slightly at the interface compared to the bulk, we have chosen to use the same cross-section scaling factor for the IR-RAS as for the ATR (with the additional surface activity correction) to calculate the degree of dissociation, given the difficulties of disentangling potential changes in cross-section with changes in surface concentration in the IR-RA spectra. We have chosen this approach to be conservative in our reports of shifts in degree of dissociation at the interface using IR-RAS.

Table S5. Ratios obtained from Avg. $A_{pH10.9}/A_{pH2.7}$ of the CH Stretches in IR-RAS of 20 mM HOA

Baseline Used in Fit	Ratio of ν_{CH3}	Ratio of $\nu_{CH2, asym}$	Ratio of $\nu_{CH2, sym}$
Logpoly5	0.66	0.73	0.69
Logcubic	0.69	0.69	0.65
Cubic	0.68	0.73	0.65

S6. Comparison of Fitting Methods for Determination of pKa from Degree of Dissociation

In Sections S4 and S5, we detail our analytical approach for both the bulk ATR and surface IR-RAS spectra. There are, however, a few potential methods that could have been used to fit these data, which we discuss below, as well as the rationale for our chosen approach.

One method for obtaining pKa from the carbonyl stretch is to plot the normalized area of the ν_{COO^-} and ν_{COOH} (or ν_{COOD}, if deuterated solvents are used) as a function of pH and fit the resulting curve to the Henderson-Hasselbalch equation. This approach has been used, for example, to extract bulk and surface pKa values from ATR and VSFG spectra of 1.4 M solutions of pyruvic acid.[14] This approach, in principle, could minimize the need to correct for differences in the absorption cross-section between the ν_{COO^-} and ν_{COOH} stretches (i.e., the need to calculate the scaling factor, F) that is required in the degree of dissociation approach we used.

However, it is equally valid to calculate pKa from the degree of dissociation.[4, 15] Our chosen fitting approach is based on previously published work that used FT-IR spectroscopy to extract pKa values for carboxylic acids[6] and was validated by determination of the bulk pKa of hexanoic acid (Figure S12), which is in reasonable agreement with the literature. In addition, we believe that there are distinct advantages to using the degree of dissociation to determine pKa for the datasets presented in this paper. Specifically, the concentrations of α-hydroxyacids used here are relatively low (50 mM HHA and 20 mM HOA) and limited by the aqueous solubility of these species, which results in relatively noisy spectra. As discussed previously, uneven baselines in IR-RA spectra are also expected due to wavelength-dependent refraction/reflectivity, as well as, potentially, minor differences in solution height and atmospheric composition. The carbonyl region also has interference with the water bending mode at ~1650 cm^{-1}. Because the calculation of the degree of dissociation essentially consists of taking a ratio between the area of the ν_{COO^-} and the ν_{COOH} for a given spectrum (or chosen baseline fit), this method minimizes the potential effects of varying baselines, while providing an internally consistent measure of protonation.

After calculating the degree of dissociation (α), we fit the data to a sigmoid to extract pKa values from the inflection point where $\alpha = 0.5$. Another possibility is to fit the data to a modified version of the Henderson-Hasselbalch equation,[6]

$$pH = pK_a + log\left(\frac{\alpha}{1-\alpha}\right),$$

as shown in Figure S17. Doing this, however, requires additional approximations. For our spectroscopic measurements, our limit of detection is such that, for the lowest and highest pH α-hydroxyacid solutions, we find experimentally that $\alpha = 0$ and $\alpha = 1$, respectively. However, in the modified Henderson-Hasselbalch equation, it is not possible to directly fit points corresponding to $\alpha = 0$ or $\alpha = 1$, and we must instead approximate the expected degree of dissociation at these points, which can significantly influence the resultant fit for small datasets.

The ability to fit all experimental data points is one reason why we, therefore, chose to directly fit the data to a sigmoid. Additionally, by using the sigmoid fit in Igor, we are also able to explicitly incorporate the uncertainty in the experimental degree of dissociation by weighting the fit with the standard deviation of each data point. Given the relatively large uncertainties in our experimental degree of dissociation, we feel that this is an important advantage to the sigmoid fit.

A comparison of the fits obtained using the weighted sigmoid fit and the modified Henderson-Hasselbalch equation for all datasets is included in Figure S17. The modified Henderson-Hasselbalch approach appears to slightly overestimate pKa values compared to fitting via a sigmoid. Importantly, however, significant differences in the relative shift between the surface pKa and the bulk pKa are not observed between the two methods. For example, for 50 mM HHA, the sigmoid fit gives a bulk pKa of 3.78 ± 0.03, while the modified Henderson-Hasselbalch gives a bulk value of 3.9, compared to the surface-pKa found by IR-RAS of 4.0 ± 0.1 and 4.2 by the modified Henderson-Hasselbalch approach. The change in pKa between the surface and the bulk (sigmoid: 0.2 ± 0.1 and H-H: 0.3) is the same within error for the two methods. Similarly, the bulk pKa of HOA using the modified Henderson-Hasselbalch approach is found to be 4.2 (sigmoid: 4.0 ± 0.1) with a surface-pKa of 4.7 (sigmoid: 4.4 ± 0.2), with a ΔpKa of 0.5 (sigmoid: 0.4 ± 0.2). We also note that the Henderson-Hasselbalch equation works best for weak acids with a pKa of ~5 or higher.[16] While the deviations caused by the assumption of weak acid behavior used in the Henderson-Hasselbalch equation are likely small compared to the relatively large experimental error for the α-hydroxyacids used here, we acknowledge this limitation for the sake of completeness.

We choose to report values obtained by fitting the degree of dissociation to a sigmoid because it requires fewer approximations than the modified Henderson-Hasselbalch approach, while also explicitly incorporating the experimental uncertainty in degree of dissociation as a weighting factor in the fit. The choice to use a sigmoid fit is also consistent with our overall approach to data analysis, which results in a lower estimate of the potential differences in protonation state at the interface.

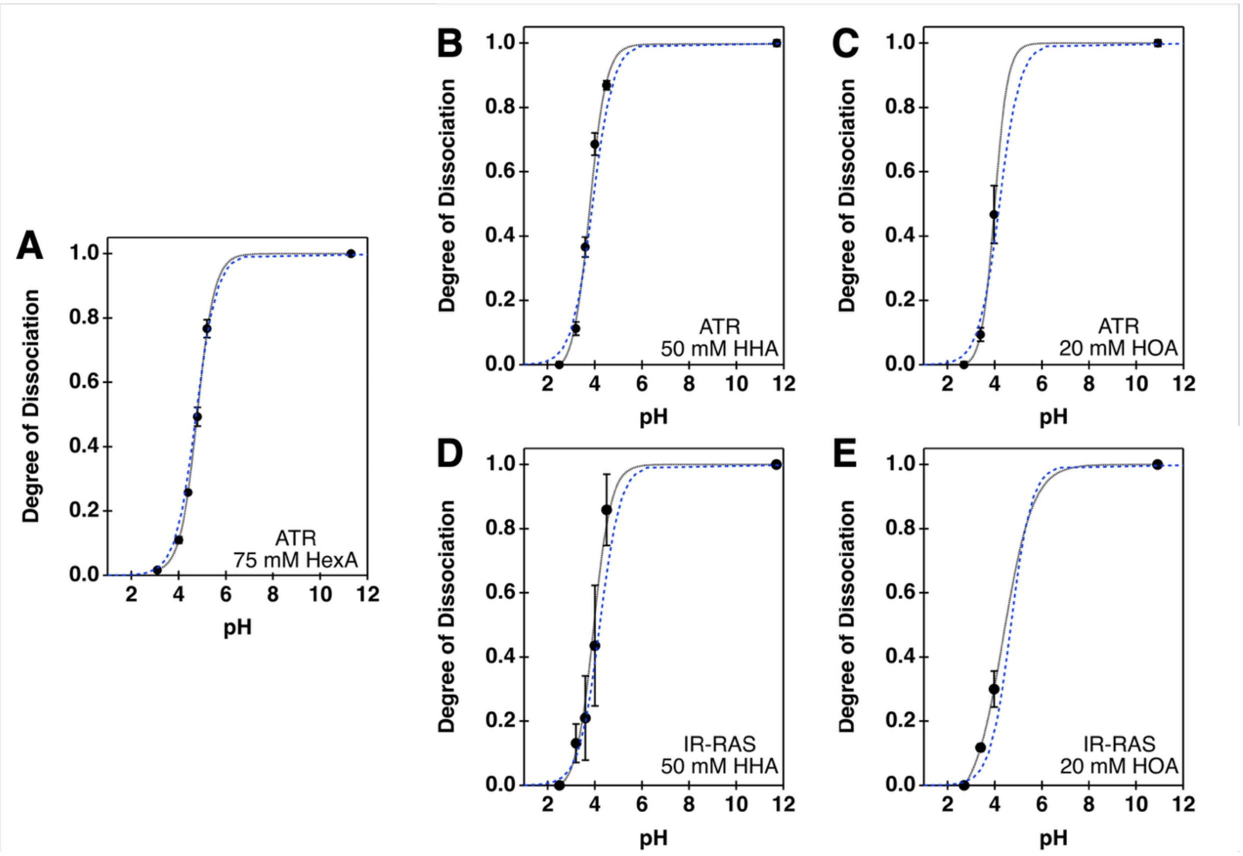

Figure S17. Comparison of fitting approaches for the extraction of pKa values from the experimental degree of dissociation (black dots) for both ATR (A-C) and IR-RAS (D-E) data for 75 mM hexanoic acid (A), 50 mM HHA (B and D), and 20 mM HOA (C and E). The black dashed lines represent the weighted sigmoid fit used to obtain the pKa values reported in the manuscript, while the blue dashed lines are fit based on the modified Henderson-Hasselbalch equation. A detailed discussion of the choice to use a sigmoid fit to determine pKa values is included in Section S6.

References:

(1) Gran, G. Determination of the equivalence point in potentiometric titrations. Part II. *Analyst* **1952**, *77* (920), 661-671. DOI: 10.1039/AN9527700661.

(2) Luo, M.; Shemesh, D.; Sullivan, M. N.; Alves, M. R.; Song, M.; Gerber, R. B.; Grassian, V. H. Impact of pH and NaCl and CaCl2 Salts on the Speciation and Photochemistry of Pyruvic Acid in the Aqueous Phase. *J. Phys. Chem. A* **2020**, *124* (25), 5071-5080. DOI: 10.1021/acs.jpca.0c01016.

(3) Luo, M.; Wauer, N. A.; Angle, K. J.; Dommer, A. C.; Song, M.; Nowak, C. M.; Amaro, R. E.; Grassian, V. H. Insights into the behavior of nonanoic acid and its conjugate base at the air/water interface through a combined experimental and theoretical approach. *Chem. Sci.* **2020**, *11* (39), 10647-10656. DOI: 10.1039/D0SC02354J.

(4) Harris, D. C. *Quantitative Chemical Analysis*; W.H. Freeman and Company, 2007.

(5) Wellen, B. A.; Lach, E. A.; Allen, H. C. Surface pKa of octanoic, nonanoic, and decanoic fatty acids at the air-water interface: Applications to atmospheric aerosol chemistry. *Phys. Chem. Chem. Phys.* **2017**, *19*, 26551-26558.

(6) Müller, M.; Wirth, L.; Urban, B. Determination of the carboxyl dissociation degree and pKa value of mono and polyacid solutions by FTIR titration. *Macromol. Chem. Phys.* **2021**, *222* (4), 2000334.
(7) Haynes, W. M. *CRC handbook of chemistry and physics*; CRC press, 2014.
(8) Cabaniss, S. E.; McVey, I. F. Aqueous infrared carboxylate absorbances: aliphatic monocarboxylates. *Spectrochimica Acta Part A: Molecular and Biomolecular Spectroscopy* **1995**, *51* (13), 2385-2395. DOI: 10.1016/0584-8539(95)01479-9.
(9) Corish, P. J.; Chapman, D. 330. The infrared spectra of some monocarboxylic acids. *Journal of the Chemical Society (Resumed)* **1957**, (0), 1746-1751. DOI: 10.1039/JR9570001746.
(10) Max, J.-J.; Chapados, C. Infrared Spectroscopy of Aqueous Carboxylic Acids: Comparison between Different Acids and Their Salts. *J. Phys. Chem. A* **2004**, *108* (16), 3324-3337. DOI: 10.1021/jp036401t.
(11) Kappes, K.; Frandsen, B. N.; Vaida, V. Infrared spectroscopy of 2-oxo-octanoic acid in multiple phases. *Phys. Chem. Chem. Phys.* **2022**, *24* (11), 6757-6768. DOI: 10.1039/d1cp05345k.
(12) Flach, C. R.; Gericke, A.; Mendelsohn, R. Quantitative Determination of Molecular Chain Tilt Angles in Monolayer Films at the Air/Water Interface: Infrared Reflection/Absorption Spectroscopy of Behenic Acid Methyl Ester. *J. Phys. Chem. B* **1997**, *101* (1), 58-65. DOI: 10.1021/jp962288d.
(13) Deal, A. M.; Vaida, V. Infrared Reflection–Absorption Spectroscopy of α-Hydroxyacids at the Water–Air Interface. *J. Phys. Chem. A* **2022**, *126* (44), 8280-8294.
(14) Lesnicki, D.; Wank, V.; Cyran, J. D.; Backus, E. H. G.; Sulpizi, M. Lower degree of dissociation of pyruvic acid at water surfaces than in bulk. *Phys. Chem. Chem. Phys.* **2022**, *24* (22), 13510-13513. DOI: 10.1039/d2cp01293f.
(15) Reijenga, J.; van Hoof, A.; van Loon, A.; Teunissen, B. Development of Methods for the Determination of pKa Values. *Analytical Chemistry Insights* **2013**, *8*, ACI.S12304. DOI: 10.4137/ACI.S12304.
(16) Po, H. N.; Senozan, N. M. The Henderson-Hasselbalch Equation: Its History and Limitations. *J. Chem. Educ.* **2001**, *78* (11), 1499. DOI: 10.1021/ed078p1499.
